# Mono- and Mixed Metal Complexes of Eu^3+^, Gd^3+^, and Tb^3+^ with a Diketone, Bearing Pyrazole Moiety and CHF_2_-Group: Structure, Color Tuning, and Kinetics of Energy Transfer between Lanthanide Ions

**DOI:** 10.3390/molecules26092655

**Published:** 2021-05-01

**Authors:** Victoria E. Gontcharenko, Mikhail A. Kiskin, Vladimir D. Dolzhenko, Vladislav M. Korshunov, Ilya V. Taydakov, Yury A. Belousov

**Affiliations:** 1 Chemistry Department, Moscow State University, Leninskie Gory, 119991 Moscow, Russia; victo.goncharenko@gmail.com (V.E.G.); doljenko_vd@inorg.chem.msu.ru (V.D.D.); 2 Kurnakov Institute of General and Inorganic Chemistry, Russian Academy of Sciences, 119991 Moscow, Russia; m_kiskin@mail.ru; 3 N.D. Zelinsky Institute of Organic Chemistry, Russian Academy of Sciences, Leninsky pr. 47, 119991 Moscow, Russia; 4 P. N. Lebedev Physical Institute of Russian Academy of Sciences, 119991 Moscow, Russia; vladkorshunov@bk.ru (V.M.K.); taidakov@mail.ru (I.V.T.); 5 Faculty of Fundamental Sciences, Bauman Moscow State Technical University, 105005 Moscow, Russia; 6 Academic Department of Innovational Materials and Technologies Chemistry, Plekhanov Russian University of Economics, 117997 Moscow, Russia

**Keywords:** pyrazoles, diketones, lanthanides, luminescence, europium, terbium, gadolinium, energy transfer, color tuning

## Abstract

Three novel lanthanide complexes with the ligand 4,4-difluoro-1-(1,5-dimethyl-1H-pyrazol-4-yl)butane-1,3-dione (HL), namely [LnL_3_(H_2_O)_2_], Ln = Eu, Gd and Tb, were synthesized, and, according to single-crystal X-ray diffraction, are isostructural. The photoluminescent properties of these compounds, as well as of three series of mixed metal complexes [Eu_x_Tb_1-x_L_3_(H_2_O)_2_] (Eu_x_Tb_1-x_L_3_), [EuxGd_1-x_L_3_(H_2_O)_2_] (Eu_x_Gd_1-x_L_3_), and [Gd_x_Tb_1-x_L_3_(H_2_O)_2_] (Gd_x_Tb_1-x_L_3_), were studied. The Eu_x_Tb_1-x_L_3_ complexes exhibit the simultaneous emission of both Eu^3+^ and Tb^3+^ ions, and the luminescence color rapidly changes from green to red upon introducing even a small fraction of Eu^3+^. A detailed analysis of the luminescence decay made it possible to determine the observed radiative lifetimes of Tb^3+^ and Eu^3+^ and estimate the rate of excitation energy transfer between these ions. For this task, a simple approximation function was proposed. The values of the energy transfer rates determined independently from the luminescence decays of terbium(III) and europium(III) ions show a good correlation.

## 1. Introduction

The unique luminescent properties of lanthanide ions are actively studied due to the possibility of their use in emitting materials [1,2,3], biovisualization [4,5,6,7], banknote protection [8] and in creating sensing materials [9,10,11].

The sensitization of the luminescence of lanthanide ions with an organic ligand has been studied since Weissman in 1942 [12] discovered sensitization of the luminescence of europium(III) ions in various chelates. This phenomenon, called the “antenna effect”, underlies the modern approach to creating Ln-based luminescent materials and allows one to avoid the problem of low-efficiency of direct excitation due to low absorption coefficients of rare-earth ions [5,13]. Various conjugated ligands, such as aromatic carboxylic acids [14,15,16], Schiff bases [17] and related compounds [18], as well as various diketones [19,20,21], have been proposed as antennae.

Among all possible chelating O,O-bidentate ligands, no doubt, 1,3-diketones are the most important and the most studied up to date. 

Lanthanide (III) diketonates have been proposed as emitting layers in OLED [22,23], as NMR shifting and discriminating agents [24], luminescent thermometers [25] and sensor materials [26]. Diketones, bearing both aromatic and perfluorinated substitutions, are widely used in coordination chemistry, chemical technology and as intermediates in organic and heterocyclic chemistry. Much less attention was paid to 1.3-diketones, which contain partially fluorinated substitutions, e.g., CHF_2_–group. Some derivatives of 4,4-difluoro-1-phenylbutane-1,3-dione (**1**) were described mainly in patents and in medicinal chemistry journals since they are important building blocks in the synthesis of Celecoxib analogs a potent cyclooxygenase-2 (COX-2) inhibitors [27,28,29]. In coordination chemistry utilizing diketones with CHF_2_–, the group is very limited. In fact, only two compounds were used as a ligands, namely, fore mentioned 4,4-difluoro-1-phenylbutane-1,3-dione (**1**), and 4,4-difluoro-1-(thiophen-2-yl)butane-1,3-dione (**2**). Complexes with Al^3+^ [30], Zr^4+^ [31], Mn^2+^ [32], Co^2+^ [33], Ni^2+^ [34] and Pd^2+^ [35] were reported up to date.

In the lanthanide series, only diketone **1** was tested as a ligand for the synthesis of Sc^3+^ [36], Tb^3+^ [37] and Eu^3+^ [38] complexes with a small set of ancillary diimine ligands.

We have recently reported unusual structural peculiarities and superb luminescent properties of lanthanide complexes with pyrazole-based 1,3-diketones, bearing perfluorinated chains of various lengths [23,39,40,41,42,43]. However, 1,3-diketones with pyrazole moieties and CHF_2_–groups have never been studied as ligands before. Here we want to report on our first results on synthesis and luminescent properties of Tb^3+^, Eu^3+^ and Gd^3+^ complexes with 1-(1,5-dimethyl-1H-pyrazol-4-yl)-4,4-difluorobutane-1,3-dione (**3**).

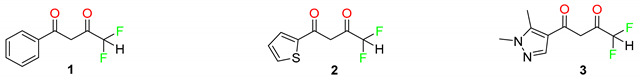



Among the many works devoted to luminescent lanthanide coordination compounds in recent years, researchers are attracted by works devoted to mixed-metal lanthanide complexes (MMLC) containing various emission centers, usually Eu^3+^ and Tb^3+^. This is facilitated by both a profound understanding of the luminescence physics of monometallic compounds and diverse applications of MMLC. Such compounds exhibit properties of “luminescent thermometers” [44,45] and also can act as chemical sensors [10,46,47] and color-tunable emitting materials [48,49,50]. The relative proximity of the excited state energies of Eu^3+^ (~17,240 cm^er1^) and Tb^3+^ (~20,400 cm^−1^) and the relatively small metal-to-metal distances in most common structures [51] cannot only provide two independent luminescence processes for each of the ions but also allow the transfer of excitation energy from Tb^3+^ ions to Eu^3+^. The thermal dependence of the efficiency of such transfer mainly determines the working mechanism of “luminescent thermometers” [52].

The existence of energy transfer in MMLC can be confirmed by the following evidence. Firstly, to provide approximately equal luminescence intensity of Eu^3+^ and Tb^3+^, the terbium(III) content in MMLC usually should exceed 90%. Second, quite often, the luminescence excitation spectra of Eu^3+^ contain peaks corresponding to the excitation of Tb^3+^ ions [10,51]. Third, introducing Eu^3+^ ions reduces the lifetimes of the excited state of terbium(III) ions significantly [52]. Finally, in a number of cases, decay curves of Eu^3+^ are not described satisfactorily by usual single- or biexponential models but contain the initial growth period [10]. Unfortunately, a detailed analysis of such kinetic curves, which can be used to determine the constant energy transfer process from Tb^3+^ to Eu^3+,^ is scarcely presented in the literature.

Compounds [LnL_3_(H_2_O)_2_], Ln = Eu, Gd, Tb, which were synthesized in this work, turned out to be convenient model objects for studying the dependence of k_ET_ on the Eu:Tb ratio in mixed-metal complexes [Eu_1-x_Tb_x_L_3_(H_2_O)_2_].

## 2. Results

### 2.1. Synthesis

The compounds were synthesized according to the common reaction of lanthanide hydroxide with an alcoholic solution of the ligand. This procedure excludes the ingress of foreign ions into the reaction mixture [20]. The composition of the compounds was confirmed by elemental analysis data (Table S1), almost complete coincidence of the IR spectra (Figures S1–S3). XRD powder patterns of all complexes coincide with the theoretical ones calculated for the europium(III), gadolinium(III) and terbium(III) complexes (Figures S4–S7).

#### Thermogravimetric Analysis

The decomposition of the Eu and Tb complexes occurs in three stages, corresponding to the peaks of the thermal effect.

At 140–165 °C, a 4% mass loss occurs for TbL_3_ with an endothermic effect, which corresponds to eliminating two water molecules from the coordination environment of lanthanide. Then, at the temperature of 200–290 °C, a loss of 29% of the sample mass with an exothermic effect is observed, which may indicate partial thermal decomposition of the ligand with water formation. Upon further heating at 300–600 °C, the mass of the sample decreases by 38.5% with intense heat release, which is explained by the ligand decomposition with the formation of water and carbon dioxide. Further heating of the sample to 700 °C does not lead to mass loss. Thus, the complex completely decomposes at 600 °C with the formation of TbF_3_ (experimental weight loss 71.5%, theoretical weight loss taking into account the formation of TbF_3_ 74.3%) and some amount of carbon (Supplementary Materials Figure S8).

The decomposition behavior of EuL_3_ is similar to the decomposition of the terbium(III) complex. However, there is a difference in the temperatures of the stages. The first stage of decomposition is observed at 160–190 °C; the second at 190–300 °C; the third at 300–560 °C. At the third stage, EuF_3_ is formed (experimental weight loss 25.7%, theoretical weight loss 25.1% (Figure S9). Thus, the TbL_3_ complex is more thermally stable.

### 2.2. Crystal Description

**Figure 1:**
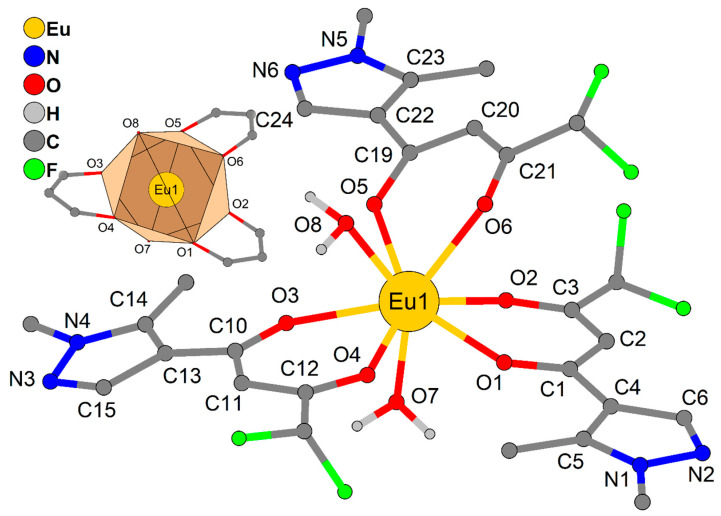
Molecular structure of **EuL_3_** (H atoms at C atoms of ligands are omitted; the inset shows the EuO_8_ polyhedron).

**Figure 2:**
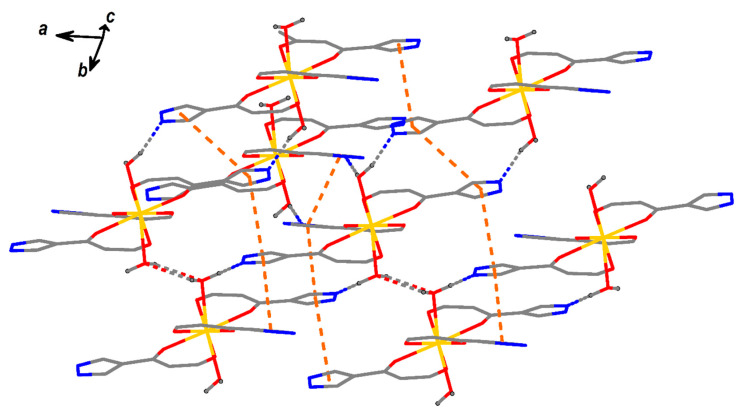
Crystal packing of **EuL_3_** (dashed lines indicate H-bonds and distances between ring centroids (Cg_I_‒Cg_J_) (for details see Table S3)).

The complexes LnL_3_ (Ln = Eu, Gd, Tb) are isostructural (Table 1). The molecules of **LnL_3_** are formed by lanthanide atom, three chelate ligands L and two water molecules (Figure 1; selected distances and angles are given in Table 2). The geometry of polyhedrons LnO_8_ corresponds to a square antiprism (see Table S2), which are formed by two planes, O1O6O8O4 and O2O5O3O7. The angles between planes of pyrazole and organometallic chelate ring (LnO_2_C_3_) are 6.5(2), 7.4(2) and 40.0(2) for **EuL_3_**, 6.4(2), 7.6(2) and 39.9(2) for **GdL_3_**, 6.3(2), 7.8(2) and 39.9(2) for **TbL_3_**. The crystal packing is due to intermolecular H-bonds, C–H…O, C–H…F, and π-π stacking (between pyrazole moieties) interactions (Figure 2, Tables S3 and S4).

### 2.3. Luminescent Properties of EuL_3_, TbL_3_ and Ln^1^_x_Ln^2^_1−x_L_3_, Ln^1^, Ln^2^=Eu, Gd, Tb

**Figure 3:**
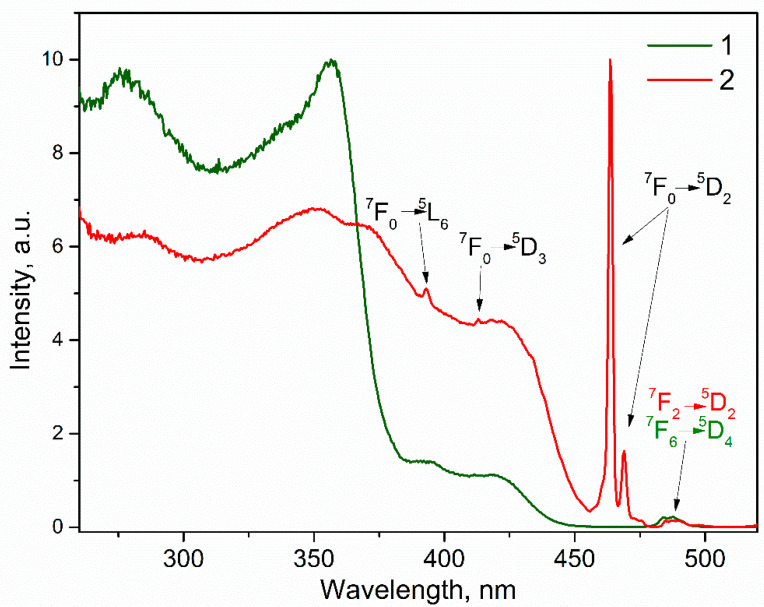
Luminescence excitation spectra recorded for complexes, with registration at 545 nm for Tb^3+^ emission (curve 1) and 617 nm for Eu^3+^ emission (curve 2).

The optical excitation spectra of luminescence consist of several broad spectral bands in the range of 260–300 nm and 310–370 nm, corresponding to π-π^*^ transitions of the ligand, as well as narrow spectral bands, which are characterized by 4f^8^ intraconfigurational transitions of ion. A band at 480–490 nm is present in the spectra of both compounds and can be attributed to ^7^F_2_–^5^D_2_ transition in the case of **EuL_3_** and ^7^F_6_–^5^D_4_ transition—in the case of **TbL_3_**. Figure 3 demonstrates the excitation spectra together with the characterization of spectral components as the f-f^*^ transitions of the ions. The excitation spectrum of **TbL_3_** scarcely contains bands associated with intraconfigurational transitions of Tb^3+^, which indicates efficient sensitization of the ionic luminescence upon excitation through the ligand. On the contrary, due to the lower energy of the ^5^D_0_ europium (III) ion resonant level compared to the ^5^D_4_ level of the Tb^3+^ ion, luminescence sensitization efficiency is less for complex **EuL_3_** than that for complex **TbL_3_** under optical excitation through ligand environment. This follows from a comparison of the emission intensities at 619 nm upon excitation through the ligand excitation bands and through the ^7^F_0_–^5^D_2_ transition of Eu^3+^. Thus, the excitation of Eu^3+^ through its ligand environment in the mixed compound will be less efficient than the excitation of Tb^3+^.

In addition, the spectra exhibit a broad band in the range of 380–450 nm, which can be attributed to the states of charge transfer between ligand molecules (LLCT), within ligand molecules (ILCT), or between the ligand and the ion (ligand to metal charge transfer, LMCT). The latter appears due to the low reduction potential of Eu^3+^ ion. Since this spectral feature is manifested for the Tb complex, which has a relatively high reduction potential, it cannot be attributed to the LMCT state.

**Figure 4:**
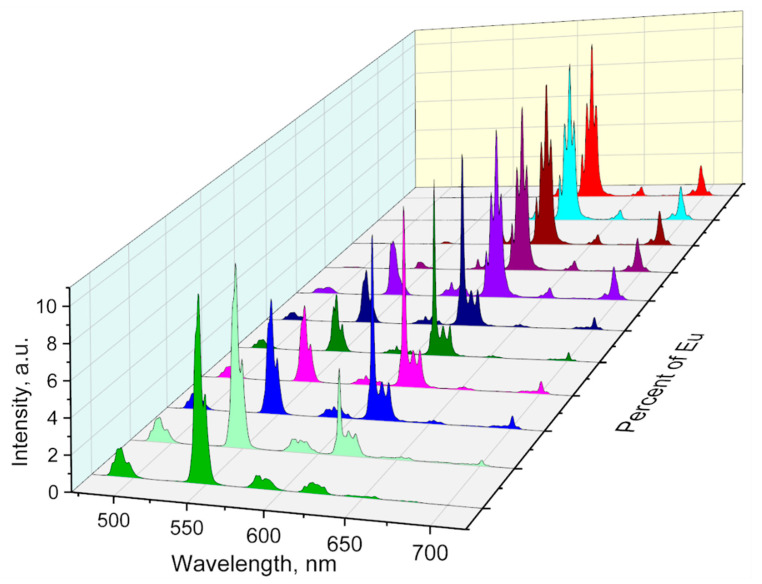
Photoluminescence spectra recorded for the investigated compounds.

The luminescence spectra of the complexes (Figure 4) contain spectral bands that are characteristic of f-f^*^ transitions of the Eu^3+^ ion: ^5^D_0_→^7^F_0_ (580 nm), ^5^D_0_→^7^F_1_ (585–590 nm), ^5^D_0_→^7^F_2_ (610–620 nm), ^5^D_0_→^7^F_3_ (650–660 nm), ^5^D_0_→^7^F_4_ (690–710 nm); as well as f-f^*^ transitions of Tb^3+^ ion: ^5^D_4_ → ^7^F_6_ (480–500 nm), ^5^D_4_ → ^7^F_5_ (535–555 nm), ^5^D_4_ → ^7^F_4_ (575–595 nm), ^5^D_4_ → ^7^F_3_ (610–630 nm), ^5^D_4_ → ^7^F_2_. (640–660 nm), ^5^D_4_ → ^7^F_1_ (660–675 nm), ^5^D_4_ → ^7^F_0_ (675–685 nm). Upon dissolving EuL_3_ and TbL_3_ in CH_3_CN no significant shifts in luminescence spectra occur (see Figure S10).

**Figure 5:**
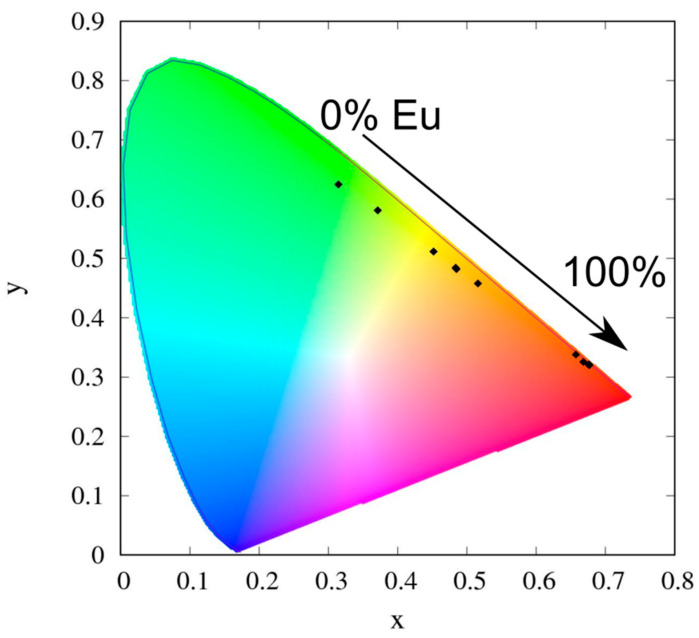
CIE chromaticity diagram.

As shown in Figure 4, with the increase in Eu concentration, the ratio of the spectral contribution Tb/Eu changes. Table 3 demonstrates that the ratio of the integrals (I_700_/I_545_) of the luminescence bands of Eu ^5^D_0_-^7^F_4_ (680–720 nm) and Tb ^5^D_4_→^7^F_5_ (535–555 nm) slightly increases from 0.10 for Eu_0.05_Tb_0.95_ L_3_ to 0.18 in the case of Eu_0.125_Tb0._875_ L_3_. Then, the ratio of the integrals increases significantly with the increasing percentage of Eu up to almost 30 in the case of Eu_0.5_Tb_0.5_ L_3_ and Eu_0.75_Tb_0.25_ L_3_. The spectral bands associated with the Eu and Tb emission have an equal number of Stark components with the same relative intensities, which indicates identical symmetry of the coordination polyhedron. Compounds Eu_0.25_Tb_0.975_ L_3_-Eu_0.125_Tb_0.875_ L_3_ are characterized by a more complex band shape in the spectral region 610–630 nm due to the contributions from the ^5^D_4_→^7^F_5_ luminescence band of Tb^3+^ and ^5^D_0_→^7^F_2_ of the Eu^3+^ ion. For the studied complexes, the chromaticity coordinates CIE were calculated (see Figure 5).

### 2.4. Description of Luminescence Decays

In the simplest case, luminescence decay is caused by the relaxation of the sole excited state and obeys the single-exponential law:

(1) $$I=I_{o}e^{-\frac{t}{\tau_{obs}}},$$

where:

(2) $$\tau_{obs}=\frac{1}{k_{obs}}=\frac{1}{k_{rad}+k_{nr}},$$

*k_rad_* and *k_nr_*—rate constants of radiative and nonradiative processes of excited state relaxation. In some cases, a deviation from single-exponential law is observed; and biexponential decay is the most common. The biexponential decay is usually explained by the presence of two independent luminescent centers.

The luminescence decay of **EuL_3_**, **TbL_3,_** as well as **Eu_1-x_Gd_x_L_3_** and **Gd_x_Tb_1-x_L_3_** can be successfully described with single-exponential curves. Rate constants k_obs_ calculated this way are shown in Table 4.

The luminescence decays of [Eu_x_Tb_1-x_L_3_(H_2_O)_2_] registered at 545 nm (Tb^3+^emission) can be well fitted by single-exponential decay. Estimated τ_obs_ of Tb^3+^ emission sharply decrease with increased Eu^3+^ concentration. It can be explained by the nonradiative energy transfer from Tb^3+^ ions to Eu^3+^ ions since the resonance energy state (^5^D_0_) of Eu^3+^ ion lies lower than the ^5^D_4_ state for Tb^3+^ ion [53].

**Figure 6:**
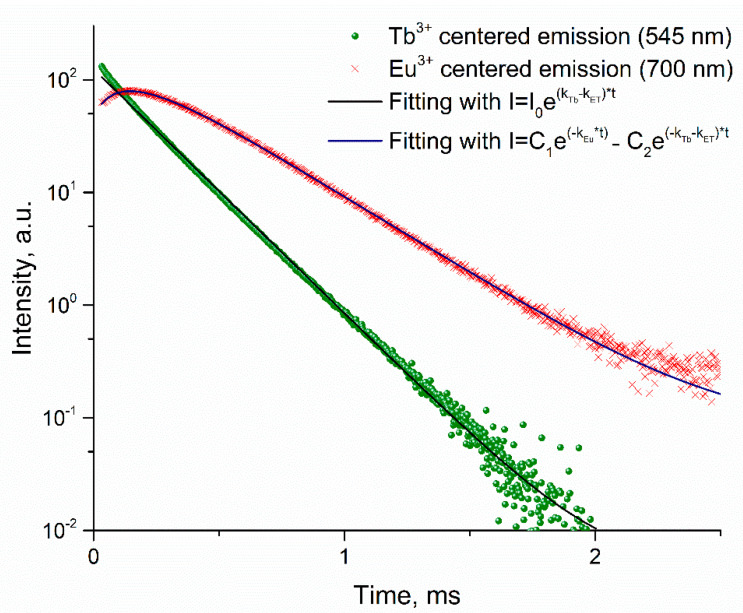
Luminescence decay of Eu^3+^ (red crosses) emission, Tb^3+^ (green bubbles) emission, and their fitting curves. Compound Eu_0.075_Tb_0.925_ L_3_, λ_EX_ 350 nm.

The typical luminescence decay curve of europium(III) emission in [Eu_x_Tb_1−x_L_3_(H_2_O)_2_] (x < 0.25) has an uncharacteristic behavior at short times, in which increased emission intensity is observed (see Figure 6).

Europium(III) luminescence decays can be described by the following model, hereinafter nonradiative relaxation of excited states of Tb^3+^ and Eu^3+^ is omitted:

(3) $$Eu^{*}\overset{k_{Eu}}{\to }Eu+h\nu_{Eu}$$

(4) $$Tb^{*}\overset{k_{Tb}}{\to }Tb+h\nu_{Tb}$$

(5) $$Tb^{*}\overset{k_{ET}}{\to }Eu^{*}$$

The decay rate of luminescence can be expressed by the following differential equations:

{(6)∂CEu*∂t=−kEuCEu*+kETCTb*(7)∂CTb*∂t=−(kTb+kET)CTb*

the solution of these equations allows one to obtain the form of the dependence of the Eu^3+^ and Tb^3+^ ions luminescence on time:

(8) $$C_{Eu^{*}}=C_{1}e^{-k_{Eu}t}+C_{2}e^{-k_{1}t},$$

(9) $${С}_{Tb^{*}}=C_{Tb^{*}}^{0}e^{-k_{1}t},$$

where:

(10) $$k_{1}=k_{ET}+k_{Tb},$$

(11) $$C_{2}=-{С}_{Tb^{*}}^{0}\frac{k_{ET}}{k_{1}-k_{Eu}},$$

(12) $$C_{1}={С}_{Eu^{*}}^{0}-C_{2}.$$

Notably, the decay of europium(III) luminescence is described by the biexponential law with a negative pre-exponential factor (*C_2_*) of the second exponential component.

As the europium(III) concentration increases, the probability of energy transfer from Tb to Eu increases, reflected in an increased *k_Eu_* constant (Table 5). At high Eu concentrations, the luminescence of terbium(III) cannot be detected because nonradiative relaxation occurs.

Observed rate constants $k_{Tb}{}^{obs}$ and $k_{Eu}{}^{obs}$ also include nonradiative relaxation processes:

(13) $$k_{Tb}{}^{obs}=k_{Tb}{}^{r}+k_{Tb}{}^{nr}$$

(14) $$k_{Eu}{}^{obs}=k_{Eu}{}^{r}+k_{Eu}{}^{nr}.$$

The energy transfer rate constant can be estimated by subtracting the k_Tb_ determined for the corresponding complex by the formula from *k_1_* (Table S9).

## 3. Materials and Methods

Lanthanide nitrates, hexahydrates (99.99%) and other reagents were purchased from Aldrich and used without additional purification. Elemental analyses were performed on the Elemental Vario MicroCube CHNO(S) analyzer. Metal content was determined by complexometric titration with Trilon B solution in the presence of Xylenol Orange as an indicator. Complexes were decomposed before analysis by heating with concentrated HNO_3_. ^1^H and ^19^ F NMR spectra were recorded on Bruker AC-300 instrument (300 and 283 MHz, respectively) at 300 K for solutions in CDCl_3_. TMS was used as an internal standard for ^1^H NMR spectra and CFCl_3_ for ^19^ F NMR spectra (δ = 0.00). ^13^C NMR spectra were recorded on a Bruker DXR-500 instrument operated at 125.8 MHz with TMS as internal standard. Mass spectra were recorded on a Thermo DSQ II/Thermo Trace instrument; the ionization energy was 70 eV (direct sample injection).

To determine the lanthanide content in mixed metal complexes, a weighted amount of the complex (about 100 mg, exact weight) was destructed in a volumetric flask with a hot concentrated nitric acid. The solution was brought to volume with double distilled water, diluted as necessary, and analyzed by ICP MS using PerkinElmer ELAN DRC-II mass spectrometer.

Photoluminescence spectra and luminescence excitation spectra were recorded at ambient temperature in the crystalline phase. For this task, a Horiba Jobin Yvon Fluorolog FL3-22 spectrofluorometer equipped with a 450 W xenon lamp emitting within the 250–900 nm spectral range was employed. The luminescence of the samples was detected with a Hamamatsu R928 photomultiplier operating within 200–850 nm. The decay kinetics of europium(III) luminescence was observed by the ^5^D_0_-^7^F_4_ transition (maximum at 700 nm) and not by the more usual intense ^5^D_0_-^7^F_2_ transition. This choice of the band for detecting luminescence is associated with the overlap of the ^5^D_0_-^7^F_2_ transition (maximum at 617 nm) with the ^5^D_4_-^7^F_3_ minor transition of terbium(III) (with a maximum at 611 nm).

Single crystal X-ray studies of crystals were carried out on a Bruker D8 Venture (for [Ln(L)_3_(H_2_O)] (Ln = Eu (EuL_3_), Tb (TbL_3_)) and Bruker D8 Quest (for [Gd(L)_3_(H_2_O)] (GdL_3_))) diffractometers equipped with a CCD detector (MoK_α_, λ = 0.71073 Å, graphite monochromator) [54]. A semiempirical adjustment for absorption was introduced for EuL_3_ and GdL_3_ [55]. Using Olex2 [56], the structures were solved with the ShelXT [57] structure solution program using Intrinsic Phasing and refined with the olex2.refine [58] refinement package using Least-Squares minimization against F^2^ in anisotropic approximation for non-hydrogen atoms. The hydrogen atoms in the ligands were calculated geometrically and refined in the “riding” model. The crystallographic parameters and the structure refinement statistics are shown in Table 1. Supplementary crystallographic data for the compounds synthesized are given in CCDC numbers 2074491 (for EuL_3_), 2074616 (for GdL_3_), 2074492 (for TbL_3_). These data can be obtained free of charge from The Cambridge Crystallographic Data Centre via www.ccdc.cam.ac.uk/data_request/cif, accessed on 24 April 2021 (CCDC: Cambridge, UK).

### 3.1. Synthesis

#### 3.1.1. Synthesis of 4,4-Difluro-1-(1,5-dimethyl-1H-pyrazol-4-yl)butane-1,3-dione (HL)

The ligand was obtained by a modified procedure described earlier [59].

Briefly, sodium hydride (4.0 g, 100 mmol, 60% dispersion in mineral oil) was placed in a 500 mL round-bottom flack under Ar blanket, and 150 mL of dry THF was added with vigorous stirring. Anhydrous EtOH (0.5 mL) was added in one portion at 0 °C, followed by the dropwise addition of a solution of 1-(1,5-dimethyl-1H-pyrazol-4-yl)ethan-1-one [60] (6.9 g, 50 mmol) and ethyl difluoroacetate (6.4 g, 51 mmol) in 30 mL of THF. When gas evolution was ceased, the cooling bath was removed, and the reaction mixture was stirred at room temperature for 15 h. After this, the dark brown solution with a small amount of precipitate was re-cooled to 0 °C, 10 mL of anhydrous EtOH was added slowly to decompose traces of NaH. The resulting solution was stirred for 30 min. The solvent was removed by evaporation under reduced pressure (100 Torr, bath temperature 40 °C), then EtOAc (40 mL) and subsequently a mixture of conc. HCl (20 mL) and crushed ice (80 mL) was added to the residue. The organic phase was separated, and the aqueous phase was then extracted with EtOAc (3 × 80 mL). The combined organic fractions were washed with brine (50 mL), dried over MgSO_4_ and evaporated to dryness. The resulting brown oil was distilled under diminished pressure. The yield was 8.76 g (81%). Yellow oil, which solidified upon standing; bp 178–180 °C (9 torr).

^1^H NMR (300 MHz CDCl_3_) δ 7.54 (s, 1H, CH=); 6.10 (s, 1H, CH); 5.91 (t, 1H, *J* = 54.5 Hz, CHF_2_); 3.83 (s, 3H, CH_3_); 2.52 (s, 3H, CH_3_). ^13^C NMR (128 Hz, CDCl_3_) δ 185.83; 175.31; 142.91; 139.45; 116.66; 109.40 (t, *J* = 245.0 Hz); 94.10 (t, *J* = 3.9 Hz); 36.12; 10.91. ^19^ F NMR (283 MHz, CDCl_3_) δ–127.8 (d, 2F, *J* = 55 Hz). LRMS *m*/*z* (%): 216 [M]^+^ (4), 165 [M–CHF_2_] ^+^ (81), 123 (81), 97 [Pyr+H]^+^ (100), 69 (32), 51 (22). Anal. calcd. for C_9_H_10_F_2_N_2_O_2_,%: C 50.00; H 4.66; N 12.96; found,%: C 50.28; H 4.63; N 12.79.

#### 3.1.2. Synthesis of Complexes

Preparation of **EuL_3_**:137.8 mg (0.309 mmol) of Eu(NO_3_)_3_·6H_2_O was dissolved in a plastic centrifuge tube with 1.5 mL of water at vigorous shaking; to precipitate Eu(OH)_3_, 200.0 μL (20% excess) of concentrated ammonia solution (25%) was added. To isolate pure Eu(OH)_3_, the solution with the precipitate was centrifuged for 2 min (8000 rpm), the solution was decanted, and the precipitate was washed with water. Centrifugation and subsequent washing of the precipitate were carried out until the smell of ammonia disappeared. Then the obtained Eu(OH)_3_ was suspended in 3 mL of EtOH and added to a solution of 200.0 mg (0.926 mmol) of the ligand in 5 mL of EtOH. The resulting mixture was heated to boiling with stirring and left overnight at room temperature. The resulting pale yellow precipitate was filtered on a vacuum pump and washed with 3 mL of diethyl ether. The yield was 83%.

Preparation of **TbL_3_**: the procedure is the same as for the synthesis of EuL_3_. A total of 200 mg (0.926 mmol) ligand and 140.0 mg (0.309 mmol) Tb(NO_3_)_3_·6H_2_O were used. The yield was 82%.

Preparation of **GdL_3_**: The procedure is the same as for the synthesis of EuL_3_. A total of 200.0 mg (0.926 mmol) ligand and 139.4 mg (0.309 mmol) Gd(NO_3_)_3_·6H_2_O were used. The yield was 78%.

The synthesis of **mixed metal complexes** was carried out according to the same procedure as for monometallic complexes. Weighed portions of Eu(NO_3_)_3_·6H_2_O, Tb(NO_3_)_3_·6H_2_O, and Gd(NO_3_)_3_·6H_2_O were taken in such a molar ratio as planned in the target compound, x = 0.01, 0.025, 0.05, 0.075, 0.10, 0.125, 0.15, 0.25, 0.5, 0.75.

The isostructurality of the compounds is confirmed by matching PXRD patterns. The Ln^1^:Ln^2^ ratio in synthesized compounds was verified by the EDX method. Thus, in Ln^1^Ln^2^_(1−x)_L_3_ compounds, various REE ions are statistically distributed. Analytical data (IR and elemental analysis data are given in the SI)

## 4. Conclusions

The molecular complexes of the HL ligand obtained in the present work demonstrate a bright luminescence of both europium (III) and terbium (III) ions. The isostructurality of europium (III), terbium (III), and gadolinium(III) compounds makes it possible to obtain mixed metal complexes with a statistical distribution of rare-earth ions, which opens up possibilities for obtaining phosphors with a tunable emission color. The use of these complexes, including mixed metal terbium–europium systems in OLED is of interest.

The proposed methodology for estimating the energy transfer constant between terbium (III) and europium (III) ions will make it possible to better describe the luminescent properties of mixed metal complexes used as luminescent thermometers and chemical sensors.

The methodology for studying the kinetics of energy transfer to the terbium-europium bimetallic complexes includes a detailed analysis (Figures S11–S14, Tables S5–S8) of the decay curve of europium (III) in the initial region where the rise to an exponential decay was proposed.

## Figures and Tables

**Table 1:** Selected crystal data and parameters for structure refinement of the Ln^3+^ complexes.

Parameter	EuL_3_	GdL_3_	TbL_3_
Empirical formula	C_27_H_31_EuF_6_N_6_O_8_	C_27_H_31_F_6_GdN_6_O_8_	C_27_H_31_F_6_TbN_6_O_8_
Formula weight	833.54	838.83	840.50
*Т* (K)	100(2)	120(2)	100(2)
Crystal system	triclinic		
Space group	*P*-1		
Crystal size (mm)	0.05 × 0.05 × 0.03	0.05 × 0.05 × 0.03	0.05 × 0.05 × 0.03
*a* (Å)	11.084(5)	11.066(2)	11.054(2)
*b* (Å)	11.968(4)	11.928(2)	11.920(2)
*c* (Å)	13.711(8)	13.673(2)	13.652(3)
α (°)	64.189(15)	64.208(3)	64.197(5)
β (°)	86.98(2)	77.956(3)	86.896(8)
γ (°)	72.669(14)	72.477(4)	72.426(5)
*V* (Å^3^)	1556.8(13)	1543.2(5)	1537.6(5)
*Z*	2	2	2
*D*_calc_ (g·cm^−3^)	1.778	1.805	1.815
*μ* (mm^−1^)	2.109	2.244	2.395
*θ* range (°)	1.95–28.28	1.95–28.0	1.94–27.48
Range of *h, k* and *l*	−14→14 −15→15 −18→16	−14→14 −13→15 0→18	−13→14 −15→14 −17→17
*Т*_min_/*Т*_max_	0.6579/0.7461	0.1806/0.3401	-
*F*(000)	832	834	836
Number of parameters	461	460	461
Reflections collected	14,998	13,219	22,779
Unique reflections	7658	7254	7041
Reflections with *I* > 2σ(*I*)	6931	6354	6311
*R*_int_	0.0304	0.0548	0.1272
*GooF*	1.062	1.077	1.014
*R*_1_(*I* > 2σ(*I*))	0.0325	0.0546	0.0350
*wR*_2_ (*I* > 2σ(*I*))	0.0679	0.1075	0.0857

**Table 2:** Selected distances and angles for **LnL_3_**.

	EuL_3_	GdL_3_	TbL_3_
Ln-O(L)	2.345 (2)-2.420 (3)	2.337 (4)-2.407 (4)	2.317 (2)-2.389 (2)
Ln-O(H_2_O)	2.445 (2), 2.536 (2)	2.419 (5)-2.518 (4)	2.412 (3), 2.510 (3)
C-O	1.259 (4)-1.278 (4)	1.258 (7)-1.272 (7)	1.261 (4)-1.279 (4)
O(L)-Eu-O(L)	71.91 (8), 72.89 (8), 73.54 (8)	72.01 (1), 73.10 (1), 73.57 (2)	72.72 (8), 73.66 (8), 74.24 (9)

**Table 3:** Calculated ratios of integrated intensities of the ^5^D_0_→^7^F_4_ Eu^3+^ transition and ^5^D_4_→^7^F_5_ Tb^3+^ transition (I_700_/I_545_) and CIE coordinates for the investigated complexes.

	2.5	5	7.5	10	12.5	15	25	50	75
I_700_/I_545_	0.02	0.10	0.14	0.18	0.40	3.09	9.02	29.46	28.07
CIE	0.37, 0.58	0.45, 0.51	0.56, 0.42	0.49, 0.48	0.48, 0.48	0.52, 0.46	0.66, 0.34	0.67, 0.33	0.68, 0.32

**Table 4:** Decay constants for Eu_x_Gd_1−x_L_3_ and Gd_x_Tb_1−x_L_3._

Compound	k_Eu_ (Eu^3+^ Decay)	Compound	k_Tb_ (Tb^3+^ Decay)
		TbL_3_	2.91
Eu_0.01_Gd_0.99_ L_3_	3.51	Gd_0.01_Tb_0.99_ L_3_	2.66
Eu_0.025_Gd_0.975_ L_3_	3.60	Gd_0.025_Tb_0.975_ L_3_	2.63
Eu_0.05_Gd_0.95_ L_3_	3.60	Gd_0.05_Tb_0.95_ L_3_	2.75
Eu_0.075_Gd_0.925_ L_3_	3.60	Gd_0.075_Tb_0.925_ L_3_	2.72
Eu_0.1_Gd_0.9_ L_3_	3.60	Gd_0.1_Tb_0.9_ L_3_	2.68
Eu_0.125_Gd_0.875_ L_3_	3.53	Gd_0.125_Tb_0.875_ L_3_	2.86
Eu_0.15_Gd_0.85_ L_3_	3.57	Gd_0.15_Tb_0.85_ L_3_	2.64
Eu_0.2_Gd_0.8_ L_3_	3.52	Gd_0.2_Tb_0.8_ L_3_	2.65
Eu_0.25_Gd_0.75_ L_3_	3.41	Gd_0.25_Tb_0.75_ L_3_	2.77
EuL_3_	3.34		

**Table 5:** Europium(III) decay and constants, calculated for Eu_x_Tb_1-x_L_3_ compounds.

Compound	k_Eu_ (Eu^3+^ Decay)	k_1_ = k_ET_ + k_Tb_. Calculated from Eu^3+^ Decay	k_1_ = k_ET_ + k_Tb_. Calculated from Tb^3+^ Decay
Eu_0.01_Tb_0.99_ L_3_	2.95	3.03	2.84
Eu_0.025_Tb_0.975_ L_3_	2.82	5.58	3.58
Eu_0.05_Tb_0.95_ L_3_	3.01	5.85	4.07
Eu_0.075_Tb_0.925_ L_3_	3.23	7.29	5.79
Eu_0.1_Tb_0.9_ L_3_	3.40	8.15	7.03
Eu_0.125_Tb_0.875_ L_3_	3.39	8.99	7.96
Eu_0.15_Tb_0.85_ L_3_	3.35	8.73	7.38
Eu_0.2_Tb_0.8_ L_3_	3.47	12.40	10.87
Eu_0.25_Tb_0.75_ L_3_	3.40	14.84	n/a

## Data Availability

Not applicable.

## Supplementary Materials

The following are available online: Table S1. CHN data and Ln1:Ln2 ratios for a mixed metal complexes Ln^1^_x_Ln^2^_1-x_L_3_, Ln^1^, Ln^2^ = Eu, Gd, Tb; Figure SI1. IR spectra of Eu_x_Gd_1-x_L_3_ complexes; Figure SI2. IR spectra of Gd_x_Tb_1-x_L_3_ complexes; Figure SI3. IR spectra of Eu_x_Tb_1-x_L_3_ complexes; Figure SI4. PXRD patterns of EuL_3_, GdL_3_ TbL_3_ and simulated from single crystal data of ones; Figure SI5. PXRD patterns of Eu_x_Gd_1-x_L_3_ compounds; Figure SI6. PXRD patterns of Gd_x_Tb_1-x_L_3_ compounds; Figure SI7. PXRD patterns of Eu_x_Tb_1-x_L_3_ compounds; Figure SI8. Mass loss (TG) and DTA curves (left) and signals from the mass spectro-metric detector of the thermal decomposition products for [TbL_3_(H_2_O)_2_] (right); Figure SI9. Mass loss (TG) and DTA curves (left) and signals from the mass spectro-metric detector of the thermal decomposition products for [EuL_3_(H_2_O)_2_] (right); Table S2. SHAPE analysis for compounds LnL_3_; Table S3. Selected parameters of intermolecular π-π interactions in LnL_3_ (CgI/J is plane of 5-memberd ring, CgI-CgJ is distance between ring centroids, α is dihedral angle between planes I and J, CgI-Perp is perpendicular distance of CgI on ring J, CgJ_Perp is perpendicular distance of CgJ on ring I, Slippage is distance between CgI and perpendicular projection of CgJ on ring I); Table S4. Selected parameters of O-H…O, O-H…N, C-H…O, and C-H…F interactions in LnL_3_; Figure SI10. Emission spectra of EuL_3_ and TbL_3_ in CH_3_CN solution; λ_EX_ = 350 nm; Figure SI11. Eu^3+^ decay curves for for Eu_x_Gd_1-x_L_3_; λ_EX_ = 350 nm, λ_EM_ = 700 nm; Table SI5. Eu^3+^ luminescence fitting parameters for Eu_x_Gd_1-x_L_3_; Figure SI12. Tb^3+^ decay curves for for Gd_x_Tb_1-x_L_3_; λ_EX_ = 350 nm, λ_EM_ = 545 nm; Table SI6. Tb^3+^ luminescence fitting parameters for Gd_x_Tb_1-x_L_3_; Figure SI13. Eu^3+^ decay curves for for Eu_x_Tb_1-x_L_3_; λ_EX_ = 350 nm, λ_EM_ = 700 nm; Table SI7. Eu^3+^ luminescence fitting parameters for Eu_x_Tb_1-x_L_3_; Figure SI14. Tb^3+^ decay curves for for Eu_x_Tb_1-x_L_3_; λ_EX_ = 350 nm, λ_EM_ = 545 nm; Table SI8. Tb^3+^ luminescence fitting parameters for Eu_x_Tb_1-x_L_3_; Table SI9. Calculated Tb^3+^ to Eu^3+^ energy transfer rate constants for Eu_x_Tb_1-x_L_3_ compounds.

## Sample Availability

Samples of the compounds are not available from the authors.
