# Supplementary material for: Mono- and Mixed Metal Complexes of Eu3+, Gd3+, and Tb3+ with a Diketone, Bearing Pyrazole Moiety and CHF2-Group: Structure, Color Tuning, and Kinetics of Energy Transfer between Lanthanide Ions
Source: Molecules. 2021 May 1;26(9):2655. doi: 10.3390/molecules26092655 (PMC8124961; doi:10.3390/molecules26092655)
Supplement: Supplementary file 1 [file molecules-26-02655-s001.zip › molecules-1187170-supplementary.pdf]

Supplementary Material

Mono- and mixed metal complexes of Eu^{III}, Gd^{III}, and Tb^{III} with a diketone, bearing pyrazole moiety and CHF_2-group : structure, color tuning, and kinetics of energy transfer between lanthanide ions

Victoria E. Gontcharenko [1], Mikhail A. Kiskin [2], Vladimir D. Dolzhenko [1,3], Vladislav M. Korshunov [4,5], Ilya V. Taydakov [4,6] and Yury A. Belousov [1,4,*]

[1] Moscow State University, Chemistry Department, Leninskie Gory, Moscow 119991, Russian Federation; victo.goncharenko@gmail.com (V.E.G.), doljenko_vd@inorg.chem.msu.ru (V.D.D.), belousov@inorg.chem.msu.ru (Yu.A.B.)
[2] Kurnakov Institute of General and Inorganic Chemistry, Russian Academy of Sciences, Moscow 119991, Russia; m_kiskin@mail.ru (M.A.K.)
[3] N.D. Zelinsky Institute of Organic Chemistry, Russian Academy of Sciences, Leninsky pr. 47, Moscow 119991, Russia
[4] P. N. Lebedev Physical Institute of Russian Academy of Sciences, Moscow 119991, Russia; vladkorshunov@bk.ru (V.M.K.), taidakov@mail.ru (I.V.T.)
[5] Bauman Moscow State Technical University, Moscow 105005, Russia
[6] G.V. Plekhanov Russian University of Economics, Moscow 117997, Russian Federation

* Correspondence: belousov@inorg.chem.msu.ru (Yu.A.B.)

Contents

Table S1. CHN data and Ln1:Ln2 ratios for a mixed metal complexes Ln1xLn21-xL3, Ln1, Ln2 = Eu, Gd, Tb 3

Figure SI1. IR spectra of EuxGd1-xL3 complexes 4

Figure SI2. IR spectra of GdxTb1-xL3 complexes 5

Figure SI3. IR spectra of EuxTb1-xL3 complexes 6

Figure SI4. PXRD patterns of EuL3, GdL3 TbL3 and simulated from single crystal data of ones. 7

Figure SI5. PXRD patterns of EuxGd1-xL3 compounds. 8

Figure SI6. PXRD patterns of GdxTb1-xL3 compounds. 9

Figure SI7. PXRD patterns of EuxTb1-xL3 compounds. 10

Figure SI8. Mass loss (TG) and DTA curves (left) and signals from the mass spectrometric detector of the thermal decomposition products for [TbL3(H2O)2] (right). 11

Figure SI9. Mass loss (TG) and DTA curves (left) and signals from the mass spectrometric detector of the thermal decomposition products for [EuL3(H2O)2] (right). 12

Table S2. SHAPE analysis for compounds LnL3. **13**

Table S3. Selected parameters of intermolecular π-π interactions in LnL3 (CgI/J is plane of 5-memberd ring, CgI-CgJ is distance between ring centroids, α is dihedral angle between planes I and J, CgI-Perp is perpendicular distance of CgI on ring J, CgJ_Perp is perpendicular distance of CgJ on ring I, Slippage is distance between CgI and perpendicular projection of CgJ on ring I). **14**

Table S4. Selected parameters of O-H…O, O-H…N, C-H…O, and C-H…F interactions in LnL3. **15**

Figure SI10. Emission spectra of EuL_3 and TbL_3 in CH_3CN solution; λ_{EX}=350 nm. **16**

Figure SI11. Eu3+ decay curves for for EuxGd1-xL3; λEX=350 nm, λEM=700 nm. **17**

Table SI5. Eu3+ luminescence fitting parameters for EuxGd1-xL3 **18-19**

Figure SI12. Tb3+ decay curves for for GdxTb1-xL3; λEX=350 nm, λEM=545 nm. **20**

Table SI6. Tb3+ luminescence fitting parameters for GdxTb1-xL3 **21-22**

Figure SI13. Eu3+ decay curves for for EuxTb1-xL3; λEX=350 nm, λEM=700 nm. **23**

Table SI7. Eu3+ luminescence fitting parameters for EuxTb1-xL3 **24-26**

Figure SI14. Tb3+ decay curves for for EuxTb1-xL3; λEX=350 nm, λEM=545 nm. **27**

Table SI8. Tb3+ luminescence fitting parameters for EuxTb1-xL3 **28-29**

Table SI9. Calculated Tb^{3+} to Eu^{3+} energy transfer rate constants for $Eu_xTb_{1-x}L_3$ compounds 30

Table S1. CHN data and Ln[1]:Ln[2] ratios for a mixed metal complexes $Ln^1_xLn^2_{1-x}L_3$, Ln[1], Ln[2] = Eu, Gd, Tb.

	C		H		N		Ln[1]:Ln[2] ratio (EDX)		Eu (ICP-MS)		Tb (ICP-MS)	
	Exp.	Calc.	Exp.	Calc.	Exp.	Calc.	Exp.	Calc.	Exp.	Calc.	Exp.	Calc.
Eu1Tb99	38.2	38.583	3.8	3.720	9.9	10.001	1:98.3	1:99	0.18	0.18	18.77	18.72
Eu2.5Tb97.5	38.2	38.588	3.6	3.721	10.0	10.002	1:40.1	1:39	0.44	0.45	18.57	18.44
Eu5Tb95	37.8	38.596	3.7	3.722	10.0	10.004	1:19.2	1:19	0.91	0.90	17.91	17.97
Eu7.5Tb92.5	37.8	38.604	3.7	3.722	10.2	10.006	1:13.5	1:12.33	1.35	1.36	17.31	17.50
Eu10Tb90	39.4	38.612	3.8	3.723	10.1	10.008	1:9.2	1:9	1.80	1.81	17.01	17.03
Eu12.5Tb87.5	39.0	38.620	3.6	3.724	10.0	10.010	1:6.8	1:7	2.24	2.26	16.52	16.56
Eu15Tb85	39.0	38.628	3.7	3.725	9.9	10.012	1:5.5	1:5.67	2.71	2.72	16.23	16.09
Eu20Tb80	38.6	38.644	3.8	3.726	10.0	10.016	1:4.0	1:4	3.63	3.62	15.21	15.15
Eu25Tb75	38.3	38.660	3.7	3.728	9.8	10.020	1:3.1	1:3	4.54	4.53	14.43	14.21
Eu50Tb50	38.0	38.740	3.8	3.735	10.1	10.040	1:1.1	1:1	9.07	9.08	9.53	9.49
Eu75Tb25	39.2	38.820	3.7	3.743	10.3	10.060	3.1:1	3:1	13.58	13.64	4.84	4.76
									Eu (ICP-MS)		Gd (ICP-MS)	
									Exp.	Calc.	Exp.	Calc.
Eu1Gd99	39.4	38.662	3.8	3.720	9.9	10.021	1:99.2	1:99	0.17	0.18	18.66	18.56
Eu2.5Gd97.5	38.3	38.666	3.8	3.721	9.8	10.022	1:39.4	1:39	0.43	0.45	18.32	18.28
Eu5Gd95	39.1	38.672	3.7	3.722	9.9	10.023	1:18.6	1:19	0.93	0.91	17.84	17.81
Eu7.5Gd92.5	37.9	38.678	3.8	3.722	10.1	10.025	1:12.9	1:12.33	1.31	1.36	17.21	17.35
Eu10Gd90	39.1	38.684	3.7	3.723	9.8	10.026	1:9.1	1:9	1.79	1.81	16.81	16.88
Eu12.5Gd87.5	39.5	38.690	3.7	3.724	10.1	10.028	1:6.7	1:7	2.31	2.27	16.40	16.42
Eu15Gd85	39.1	38.696	3.7	3.725	10.2	10.029	1:5.2	1:5.67	2.75	2.72	15.75	15.95
Eu25Gd75	39.5	38.720	3.7	3.728	10.0	10.035	1:3.1	1:3	4.50	4.54	14.01	14.08
Eu50Gd50	38.0	38.780	3.8	3.735	10.3	10.050	1:1.1	1:1	9.09	9.09	9.41	9.40
Eu75Gd25	39.6	38.840	3.8	3.743	10.0	10.065	3:1.1	3:1	13.45	13.65	4.65	4.71
									Gd (ICP-MS)		Tb (ICP-MS)	
									Exp.	Calc.	Exp.	Calc.
Gd1Tb99	39.7	38.577	3.8	3.725	10.1	10.000	1:99.2	1:99	0.20	0.19	18.72	18.72
Gd2.5Tb97.5	38.6	38.582	3.8	3.720	9.8	10.001	1:38.7	1:39	0.46	0.47	18.39	18.44
Gd5Tb95	38.2	38.584	3.7	3.720	10.0	10.001	1:19.4	1:19	0.93	0.94	17.68	17.96
Gd7.5Tb92.5	38.6	38.586	3.7	3.720	10.0	10.002	1:12.2	1:12.33	1.42	1.40	17.53	17.49
Gd10Tb90	37.8	38.588	3.7	3.720	9.9	10.002	1:9.4	1:9	1.86	1.87	17.03	17.02
Gd12.5Tb87.5	38.6	38.590	3.7	3.720	10.2	10.003	1:7.1	1:7	2.44	2.34	16.54	16.56
Gd15Tb85	37.8	38.592	3.8	3.720	9.9	10.003	1:5.1	1:5.67	2.79	2.81	16.07	16.08
Gd20Tb80	37.8	38.596	3.8	3.720	10.0	10.004	1:3.9	1:4	3.78	3.74	15.33	15.13
Gd25Tb75	38.6	38.600	3.8	3.720	10.2	10.005	1:3.2	1:3	4.65	4.68	14.23	14.19
Gd50Tb50	38.2	38.620	3.6	3.720	9.9	10.010	1:0.98	1:1	9.44	9.36	9.48	9.46
Gd75Tb25	37.9	38.640	3.7	3.720	10.2	10.015	3:0.97	3:1	14.23	14.05	4.69	4.73

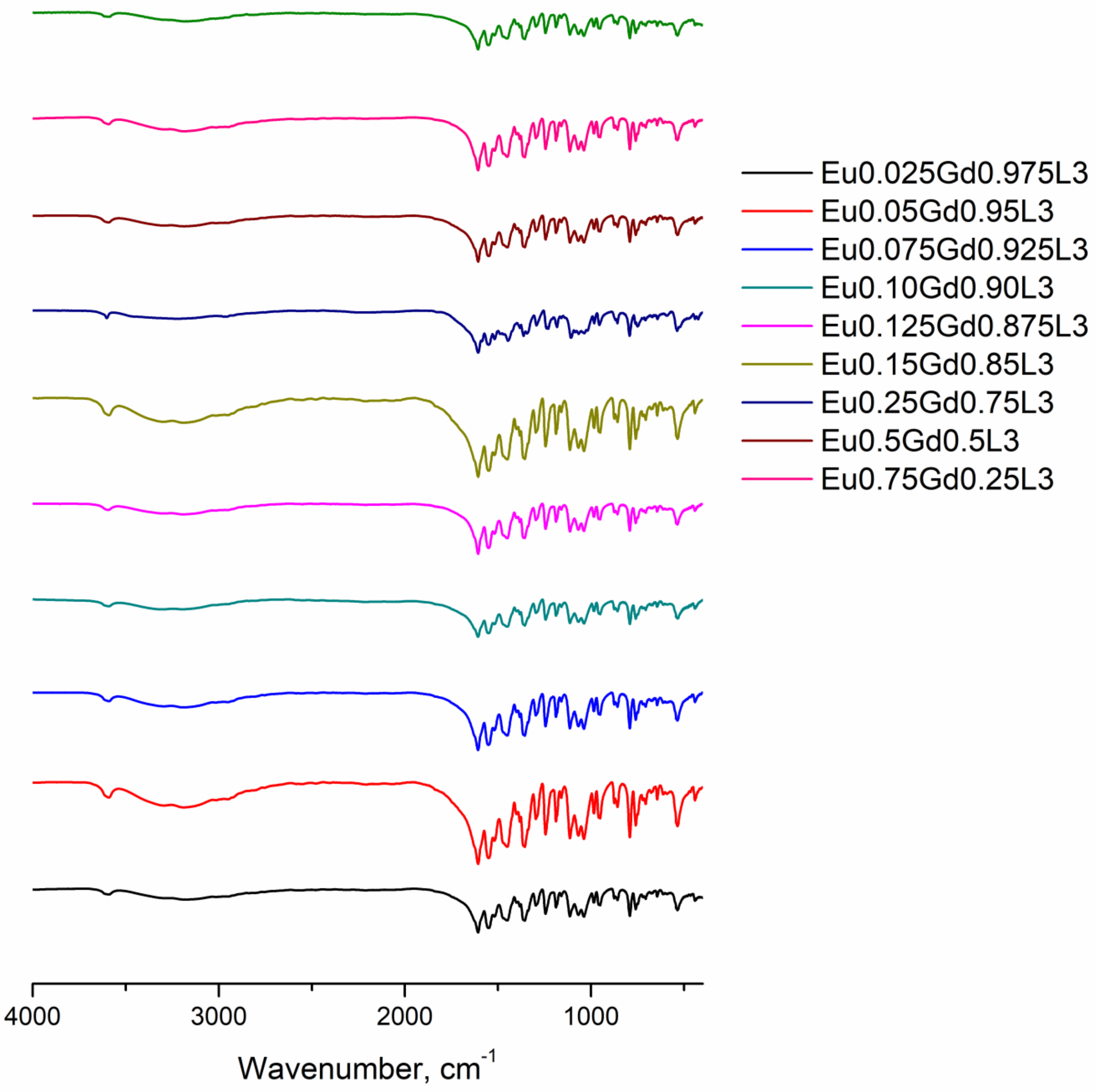

Figure SI1. IR spectra of $Eu_xGd_{1-x}L_3$ complexes

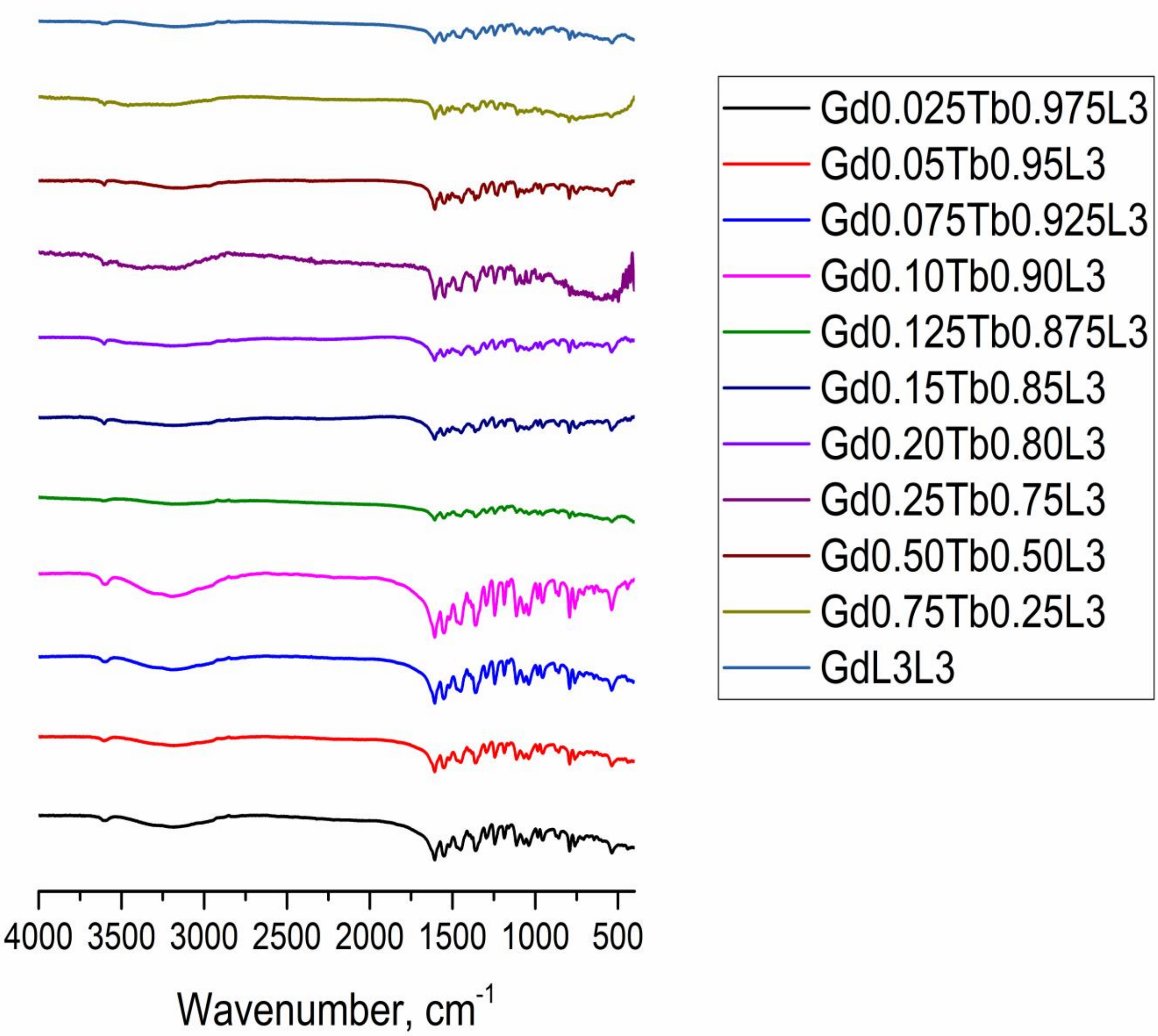

Figure SI2. IR spectra of $Gd_xTb_{1-x}L_3$ complexes

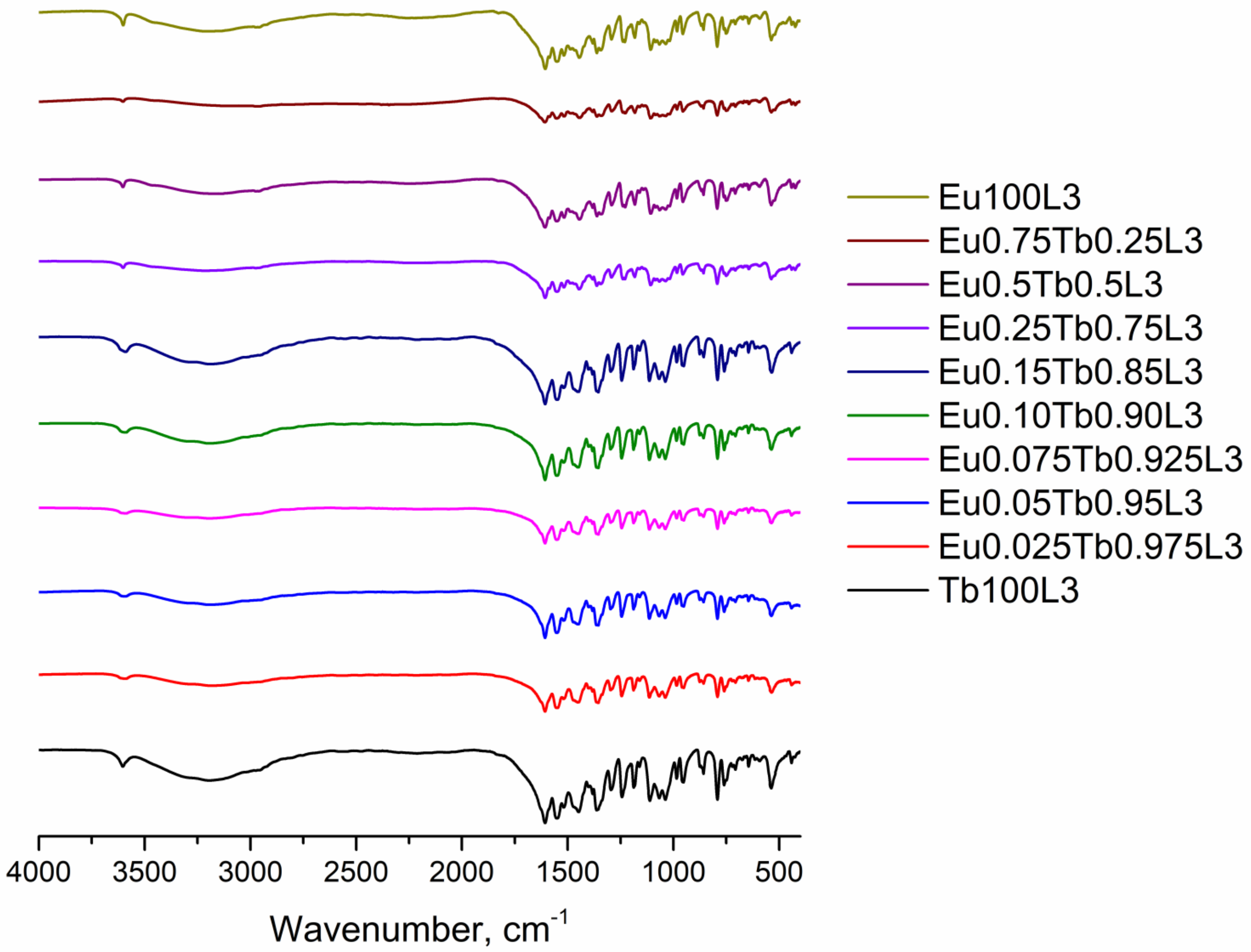

Figure SI3. IR spectra of $Eu_xTb_{1-x}L_3$ complexes

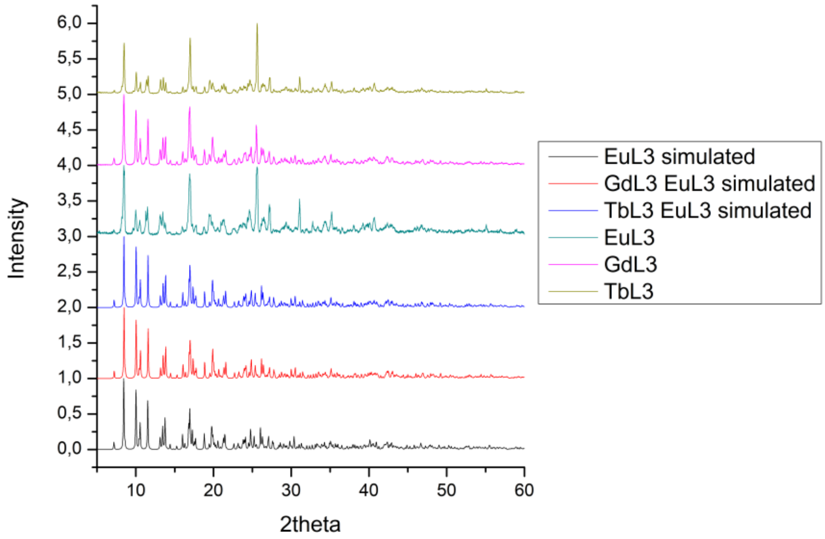

Figure SI4. PXRD patterns of EuL_3, GdL_3 TbL_3 and simulated from single crystal data of ones.

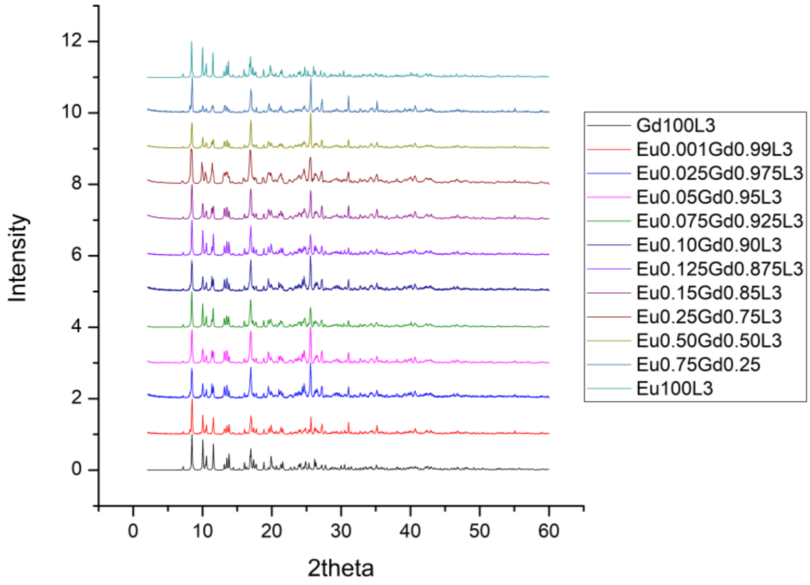

Figure SI5. PXRD patterns of $Eu_xGd_{1-x}L_3$ compounds.

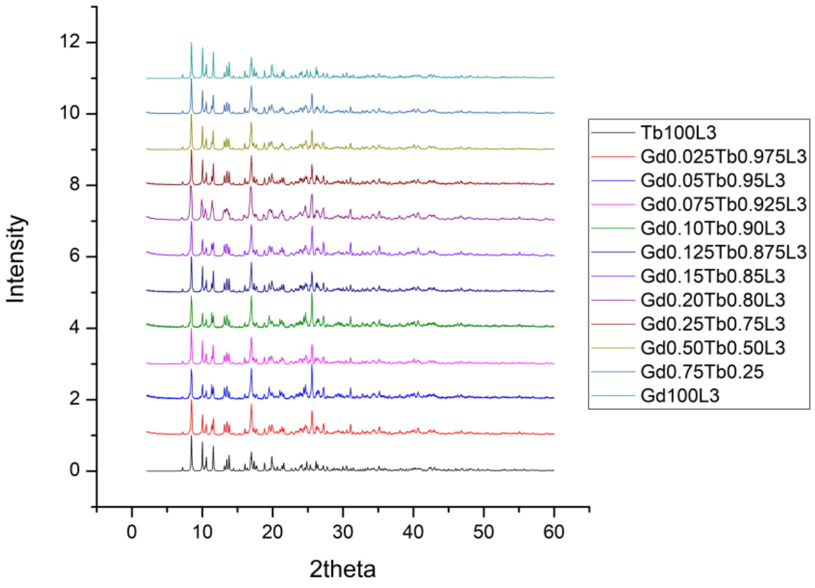

Figure SI6. PXRD patterns of $Gd_xTb_{1-x}L_3$ compounds.

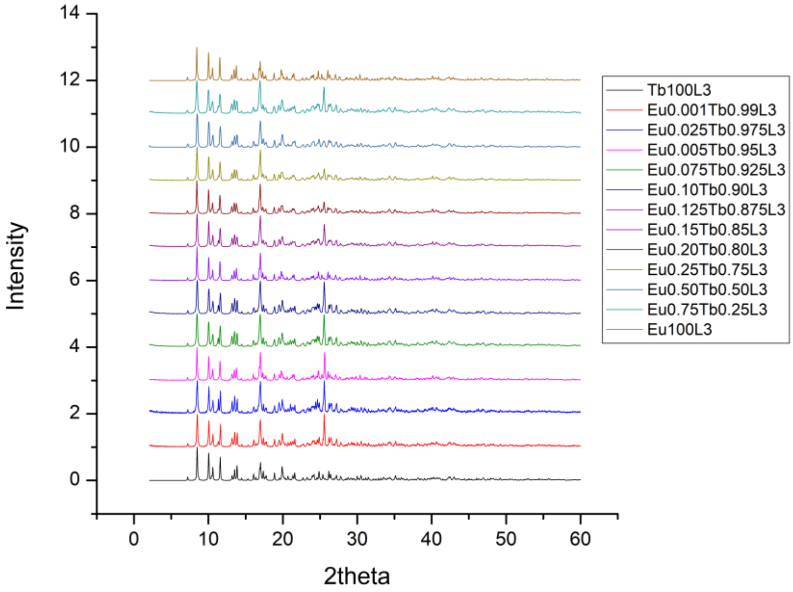

Figure SI7. PXRD patterns of $Eu_xTb_{1-x}L_3$ compounds.

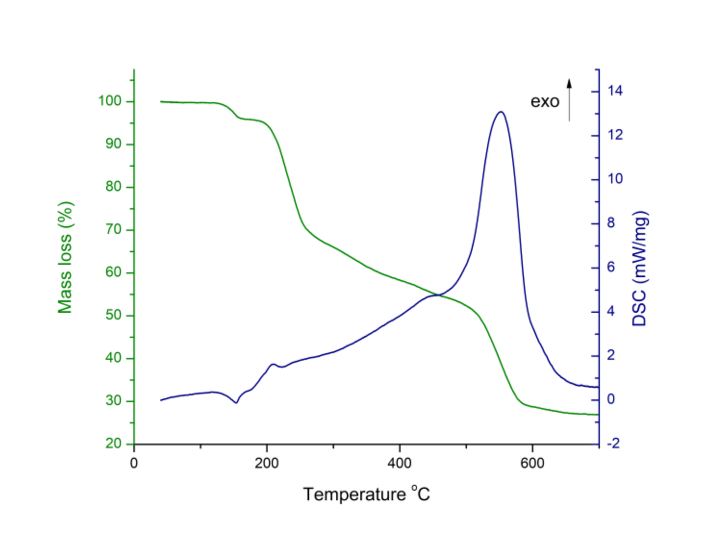

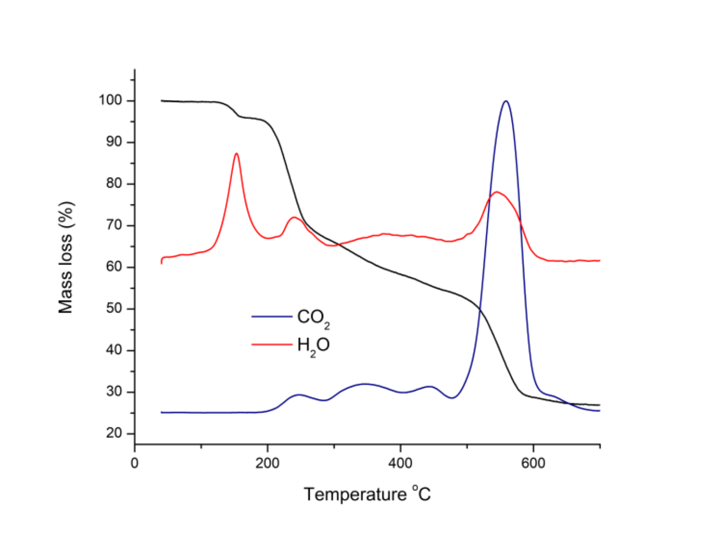

Figure SI8. Mass loss (TG) and DTA curves (left) and signals from the mass spectrometric detector of the thermal decomposition products for [TbL$_3$(H$_2$O)$_2$] (right).

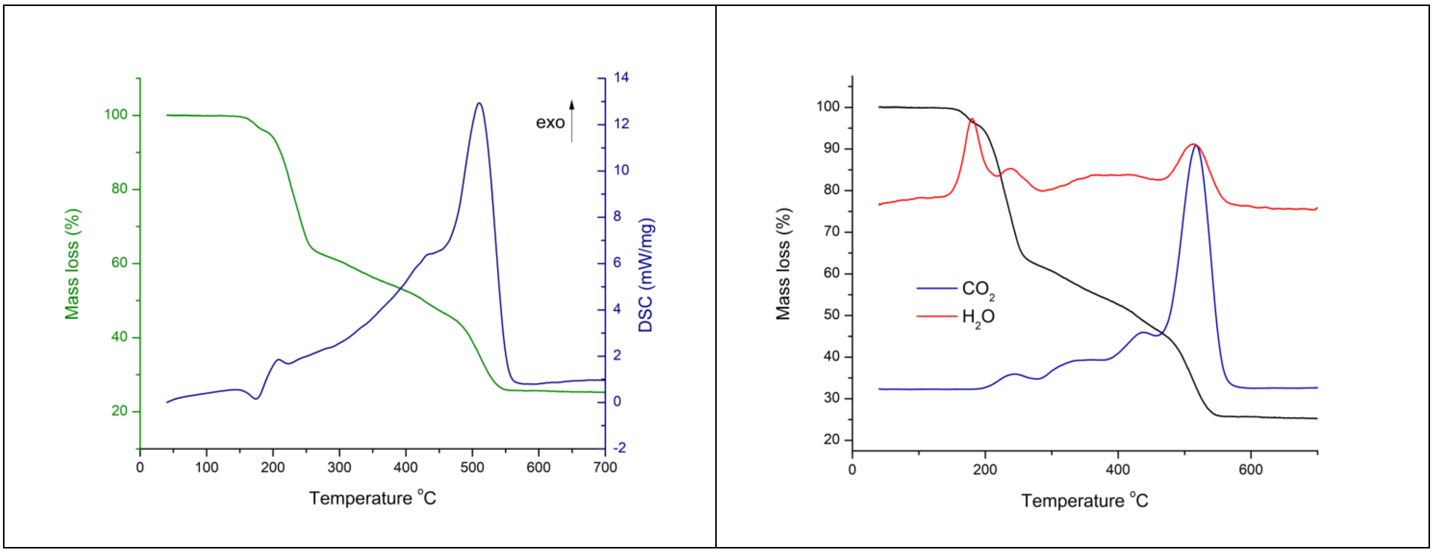

Figure SI9. Mass loss (TG) and DTA curves (left) and signals from the mass spectrometric detector of the thermal decomposition products for [EuL$_3$(H$_2$O)$_2$] (right).

Table S2. SHAPE analysis for compounds LnL_3.

	SAPR-8, D_{4d}, Square antiprism	TDD-8, D_{2d}, Triangular dodecahedron	JBTPR-8, C_{2v}, Biaugmented trigonal prism J50	BTPR-8, C_{2v}, Biaugmented trigonal prism	JSD-8, D_{2d}, Snub diphenoid J84
**1**	1.202	1.744	2.214	1.512	4.295
**2**	1.152	1.707	2.164	1.495	4.238
**3**	1.027	1.730	2.213	1.510	4.386

Table S3. Selected parameters of intermolecular π-π interactions in LnL_3 ($Cg_{I/J}$ is plane of 5-memberd ring, Cg_I-Cg_J is distance between ring centroids, α is dihedral angle between planes I and J, Cg_I-Perp is perpendicular distance of Cg_I on ring J, Cg_J_Perp is perpendicular distance of Cg_J on ring I, Slippage is distance between Cg_I and perpendicular projection of Cg_J on ring I).

Cg_I/Cg_J [symmetry code]	Cg_I-Cg_J, Å	α, deg.	Cg_I-Perp/Cg_J_Perp, Å	Slippage, Å
**EuL_3**				
N1N2C4-C6/ N1N2C4-C6 [1-x, -y, 2-z]	4.494(3)	0	3.467(2)/3.467(2)	2.859
N1N2C4-C6/ N5N6C22-C24 [-1+x, y, z]	4.015(3)	7.3(2)	3.645(2)/3.424(2)	2.096
N3N4C13-C15/ N3N4C13-C15 [2-x, -y, 1-z]	3.721(3)	0	3.439(2)/3.439(2)	1.422
N3N4C13-C15/ N5N6C22-C24 [2-x, 1-y, 1-z]	3.859(3)	15.8(2)	3.748(2)/3.404(2)	1.817
**GdL_3**				
N1N2C4-C6/N1N2C4-C6 [1-x, 1-y, -z]	4.467(4)	0	3.443(3)/3.444(3)	2.845
N1N2C4-C6/N5N6C22-C24 [-1+x, y, z]	4.002(4)	7.1(4)	3.625(3)/3.406(3)	2.102
N3N4C13-C15/N3N4C13-C15 [2-x, -y, 1-z]	3.706(4)	0	3.417(3)/3.417(3)	1.435
N3N4C13-C15/N5N6C22-C24 [2-x, 1-y, 1-z]	3.841(4)	15.8(4)	3.729(3)/3.390(3)	1.806
**TbL_3**				
N1N2C4-C6/ N1N2C4-C6 [1-x, -y, 2-z]	4.419(3)	0	3.438(2)/3.438(2)	2.777
N1N2C4-C6/ N5N6C22-C24 [-1+x, y, z]	4.016(3)	7.1(3)	3.631(2)/3.406(2)	2.127
N3N4C13-C15/ N3N4C13-C15 [2-x, -y, 1-z]	3.710(3)	0	3.415(2)/3.415(2)	1.449
N3N4C13-C15/ N5N6C22-C24 [2-x, 1-y, 1-z]	3.839(3)	15.7(3)	3.729(2)/3.401(2)	1.781

Table S4. Selected parameters of O-H…O, O-H…N, C-H…O, and C-H…F interactions in LnL_3.

D-H…A [symmetry index]	D-H, Å	H…A, Å	D-H…A, Å	D-H-A, deg
**EuL_3**				
O7-H7A...N3 [2-x, -y, 1-z]	0.89	1.98	2.816(4)	155
O7-H7B...N2 [1-x, -y, 2-z]	0.89	1.94	2.810(4)	166
O8-H8A...O8 [1-x, 1-y, 1-z]	0.93	2.38	2.911(4)	116
O8-H8B...N6 [2-x, 1-y, 1-z]	0.93	2.01	2.804(4)	142
C26-H26A...O6 [2-x, 1-y, 1-z]	0.98	2.58	3.375(5)	139
C15-H15...F2 [x, y, -1+z]	0.95	2.43	3.260(4)	146
C16-H16C...F6A [1+x, y, z]	0.95	2.46	3.198(6)	132
C27-H27...F5 [1-x, 1-y, 1-z]	1.00	2.30	3.065(6)	132
**GdL_3**				
O7-H7A...N3 [2-x, -y, 1-z]	0.87	1.98	2.798(7)	154
O7-H7B...N2 [1-x, 1-y, z]	0.87	1.98	2.802(7)	156
O8-H8A...N6 [2-x, 1-y, 1-z]	0.87	1.95	2.799(7)	166
O8-H8B...F4 [1-x, 1-y, 1-z]	0.87	2.03	2.893(9)	171
C6-H6…F5 [1-x, 2-y, -z]	0.95	2.62	3.219(8)	122
C15-H15…F1 [x, y-1, 1+z]	0.95	2.43	3.255(7)	145
C16-H16A…F4 [1+x, y, z]	0.98	2.46	3.207(10)	133
C25-H26C…O6 [1+x, y, z]	0.98	2.56	3.373(8)	141
C8-H8C…O2 [x-1, y, z]	0.98	2.64	3.313(8)	126
**TbL_3**				
O7-H7A...N3 [2-x, -y, 1-z]	0.89	1.97	2.802(5)	155
O7-H7B...N2 [1-x, -y, 2-z]	0.89	1.93	2.799(4)	166
O8-H8A...O8 [1-x, 1-y, 1-z]	1.00	2.37	2.913(3)	113
O8-H8B...N6 [2-x, 1-y, 1-z]	1.00	1.96	2.799(4)	140
C26-H26A...O6 [2-x, 1-y, 1-z]	0.98	2.58	3.385(4)	139
C15-H15...F2 [x, y, -1+z]	0.95	2.42	3.247(5)	146
C16-H16C...F6A [1+x, y, z]	0.95	2.45	3.194(7)	133
C27-H27...F5 [1-x, 1-y, 1-z]	0.95	2.30	3.056(6)	132

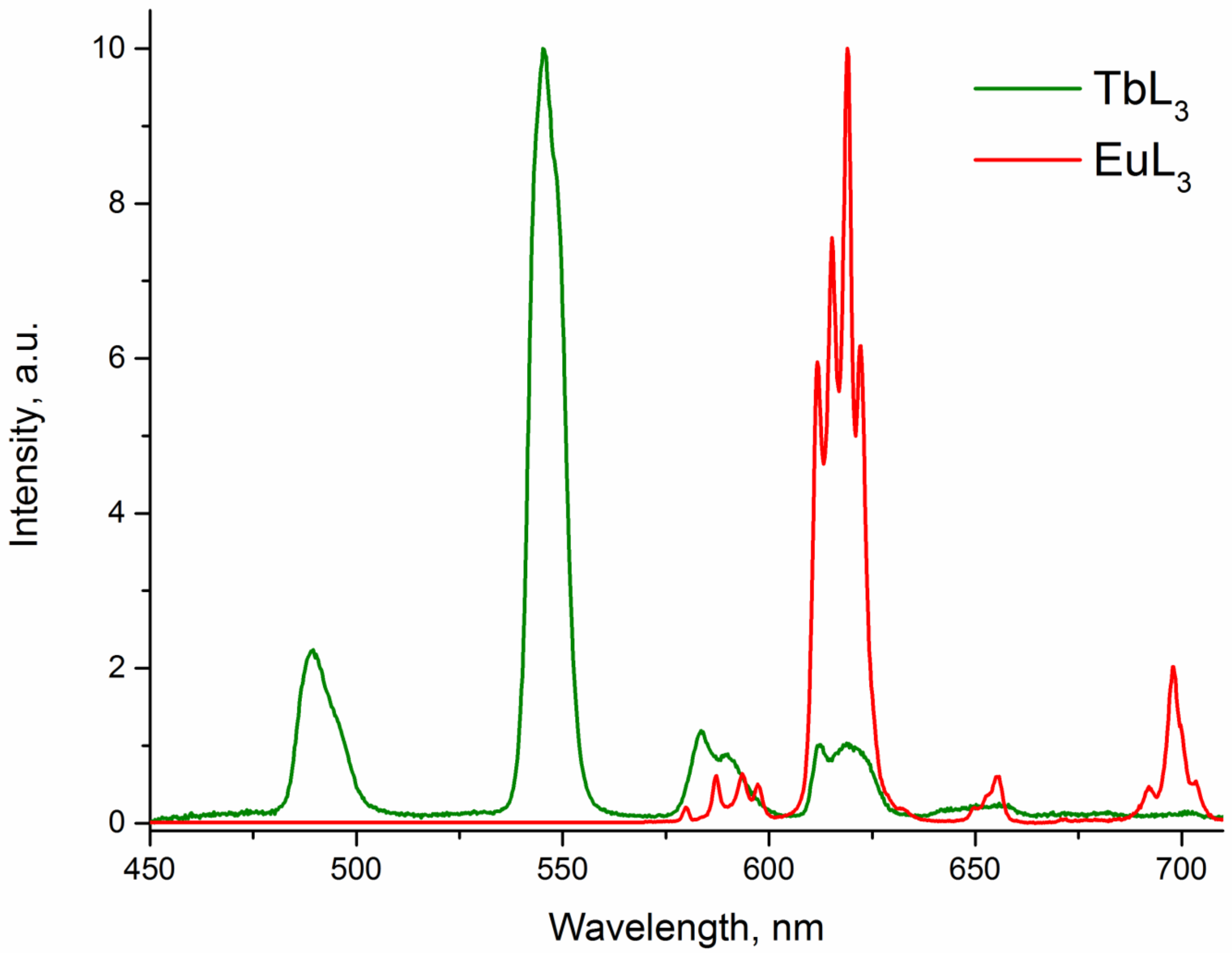

Figure SI10. Emission spectra of EuL_3 and TbL_3 in CH_3CN solution; λ_{EX}=350 nm.

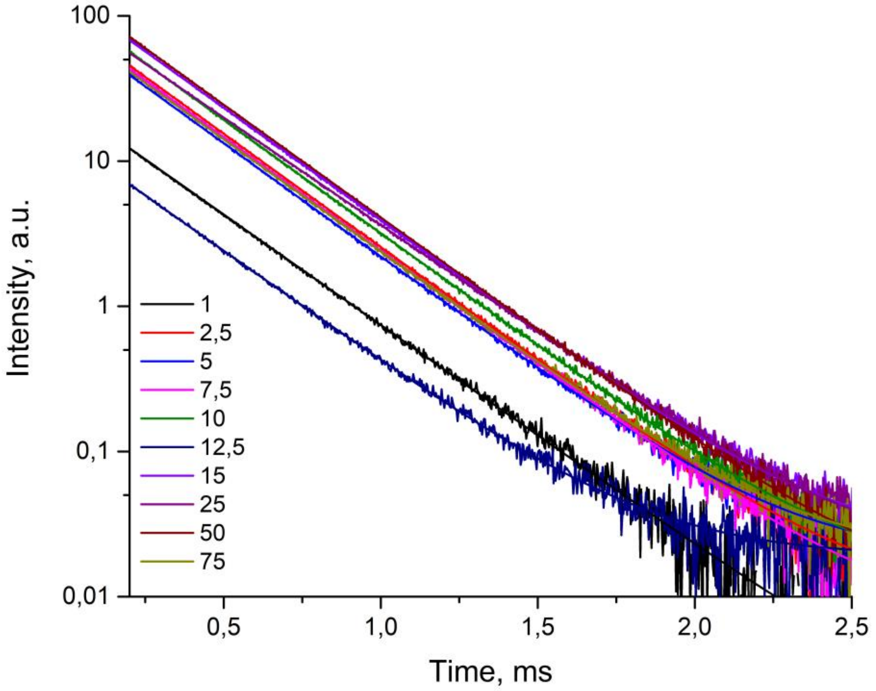

Figure SI11. Eu^{3+} decay curves for for $Eu_xGd_{1-x}L_3$; λ_{EX}=350 nm, λ_{EM}=700 nm.

Table SI5. Eu^{3+} luminescence fitting parameters for $Eu_xGd_{1-x}L_3$

% Eu	Equation : y = A1*exp(-x/t1) + y0	Value	Standart error
1	Reduced Chi-Sqr	1.18E-04	
1	Adj. R-Square	0.9999	
1	y0	9.98E-04	5.38E-04
1	A1	24.75768	0.09189
1	t1	0.28507	4.21E-04
1	k	3.5079	0.00518
2.5	Reduced Chi-Sqr	5.05E-05	
2.5	Adj. R-Square	0.9999	
2.5	y0	0.00972	3.93E-04
2.5	A1	93.18534	0.21079
2.5	t1	0.27785	1.96E-04
2.5	k	3.59905	0.00254
5	Reduced Chi-Sqr	5.30E-05	
5	Adj. R-Square	0.9999	
5	y0	0.01854	3.96E-04
5	A1	80.28002	0.18866
5	t1	0.27744	2.08E-04
5	k	3.60438	0.00271
7.5	Reduced Chi-Sqr	5.12E-05	
7.5	Adj. R-Square	0.9999	
7.5	y0	0.00689	4.09E-04
7.5	A1	88.59189	0.21102
7.5	t1	0.27766	2.08E-04
7.5	k	3.60158	0.0027
10	Reduced Chi-Sqr	5.54E-05	
10	Adj. R-Square	0.9999	
10	y0	0.01401	4.06E-04
10	A1	116.1669	0.26066
10	t1	0.27779	1.88E-04
10	k	3.59986	0.00244
12.5	Reduced Chi-Sqr	7.06E-05	
12.5	Adj. R-Square	0.9999	
12.5	y0	0.01897	3.97E-04
12.5	A1	13.9579	0.04294
12.5	t1	0.28354	3.91E-04
12.5	k	3.52678	0.00486
15	Reduced Chi-Sqr	6.80E-05	
15	Adj. R-Square	0.9999	
15	y0	0.02291	4.82E-04
15	A1	138.441	0.34866
15	t1	0.28048	2.08E-04
15	k	3.56534	0.00265

25	Reduced Chi-Sqr	5.68E-05	
25	Adj. R-Square	0.9999	
25	y0	0.01842	4.47E-04
25	A1	109.7018	0.24253
25	t1	0.29295	2.01E-04
25	k	3.41354	0.00235
50	Reduced Chi-Sqr	5.25E-05	
50	Adj. R-Square	0.9999	
50	y0	0.00953	4.25E-04
50	A1	146.1477	0.32402
50	t1	0.27945	1.81E-04
50	k	3.57841	0.00232
75	Reduced Chi-Sqr	5.95E-05	
75	Adj. R-Square	0.9999	
75	y0	0.01866	4.25E-04
75	A1	84.00853	0.20549
75	t1	0.28033	2.18E-04
75	k	3.56717	0.00278

.

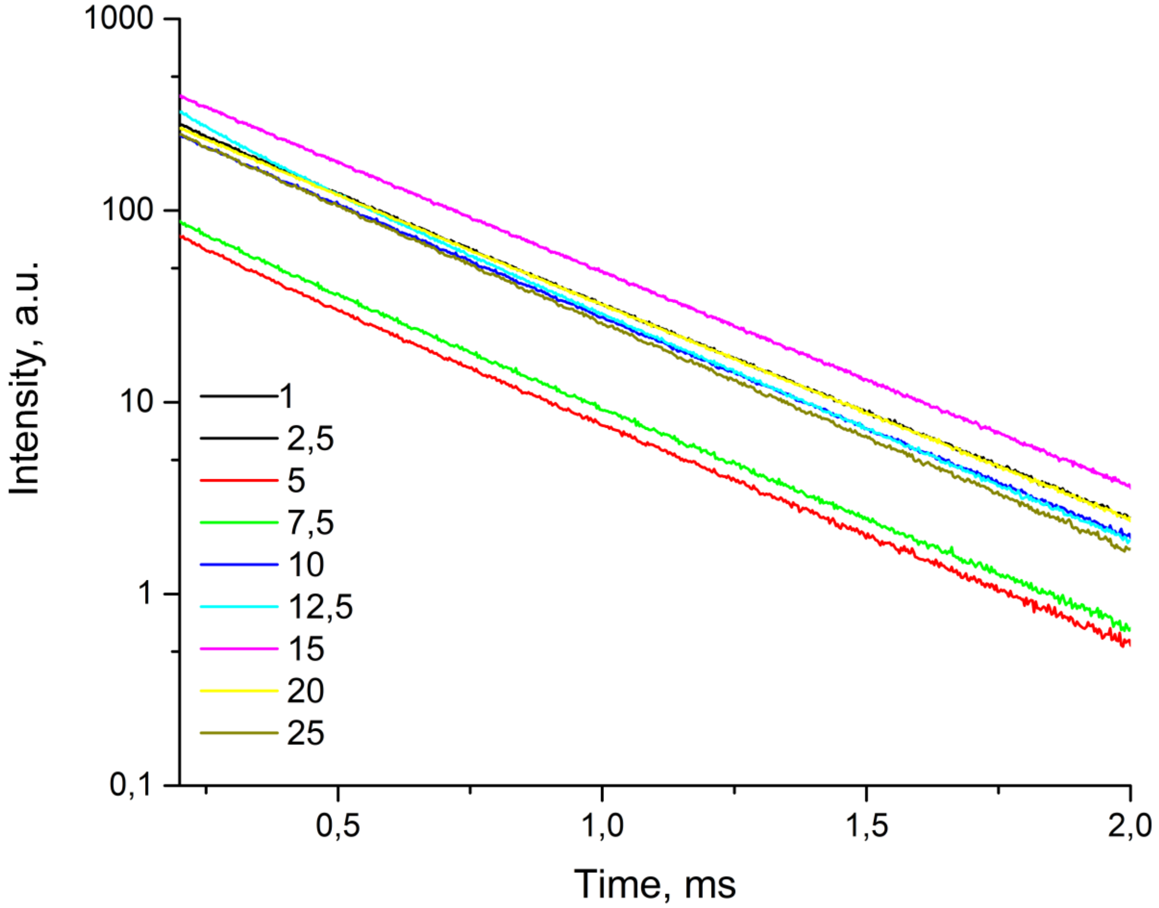

Figure SI12. Tb^{3+} decay curves for for $Gd_xTb_{1-x}L_3$; λ_{EX}=350 nm, λ_{EM}=545 nm.

Table SI6. Tb^{3+} luminescence fitting parameters for $Gd_xTb_{1-x}L_3$

% Gd	Equation : y = A1*exp(-x/t1) + y0	Value	Standart error
1	Reduced Chi-Sqr	1.28E-4	
1	Adj. R-Square	0.998	
1	y0	0.061	0.00261
1	A1	572.2	1.0023
1	t1	0.3759	2.65683E-4
1	k	2.6604	0.00158
2.5	Reduced Chi-Sqr	1.19E-04	
2.5	Adj. R-Square	0.999	
2.5	y0	0.062	0.00165
2.5	A1	461.30764	1.00823
2.5	t1	0.38039	2.54353E-4
2.5	k	2.62885	0.00176
5	Reduced Chi-Sqr	2.16E-04	
5	Adj. R-Square	0.999	
5	y0	0.03471	0.0012
5	A1	122.42287	0.40495
5	t1	0.36382	4.07121E-4
5	k	2.7486	0.00308
7.5	Reduced Chi-Sqr	1.91E-04	
7.5	Adj. R-Square	0.999	
7.5	y0	0.03073	0.00121
7.5	A1	144.61627	0.44121
7.5	t1	0.36714	3.73217E-4
7.5	k	2.72374	0.00277
10	Reduced Chi-Sqr	1.14E-04	
10	Adj. R-Square	0.999	
10	y0	0.03503	0.00142
10	A1	412.85	0.90735
10	t1	0.37276	2.48418E-4
10	k	2.68267	0.00179
12.5	Reduced Chi-Sqr	5.63E-04	
12.5	Adj. R-Square	0.998	
12.5	y0	0.0678	0.00287
12.5	A1	534.02008	2.76546
12.5	t1	0.35002	5.08617E-4
12.5	k	2.85694	0.00415
15	Reduced Chi-Sqr	1.68E-05	
15	Adj. R-Square	0.999	
15	y0	0.25724	0.00901
15	A1	667.76301	0.56589

15	t1	0.37927	1.6378E-4
15	k	2.63661	0.00114
20	Reduced Chi-Sqr	1.65E-05	
20	Adj. R-Square	0.997	
20	y0	0.20149	0.00596
20	A1	455.60949	0.38478
20	t1	0.37739	1.61141E-4
20	k	2.64976	0.00113
25	Reduced Chi-Sqr	1.15E-04	
25	Adj. R-Square	0.999	
25	y0	0.02395	0.00129
25	A1	423.55277	0.96991
25	t1	0.36041	2.42923E-4
25	k	2.77461	0.00187

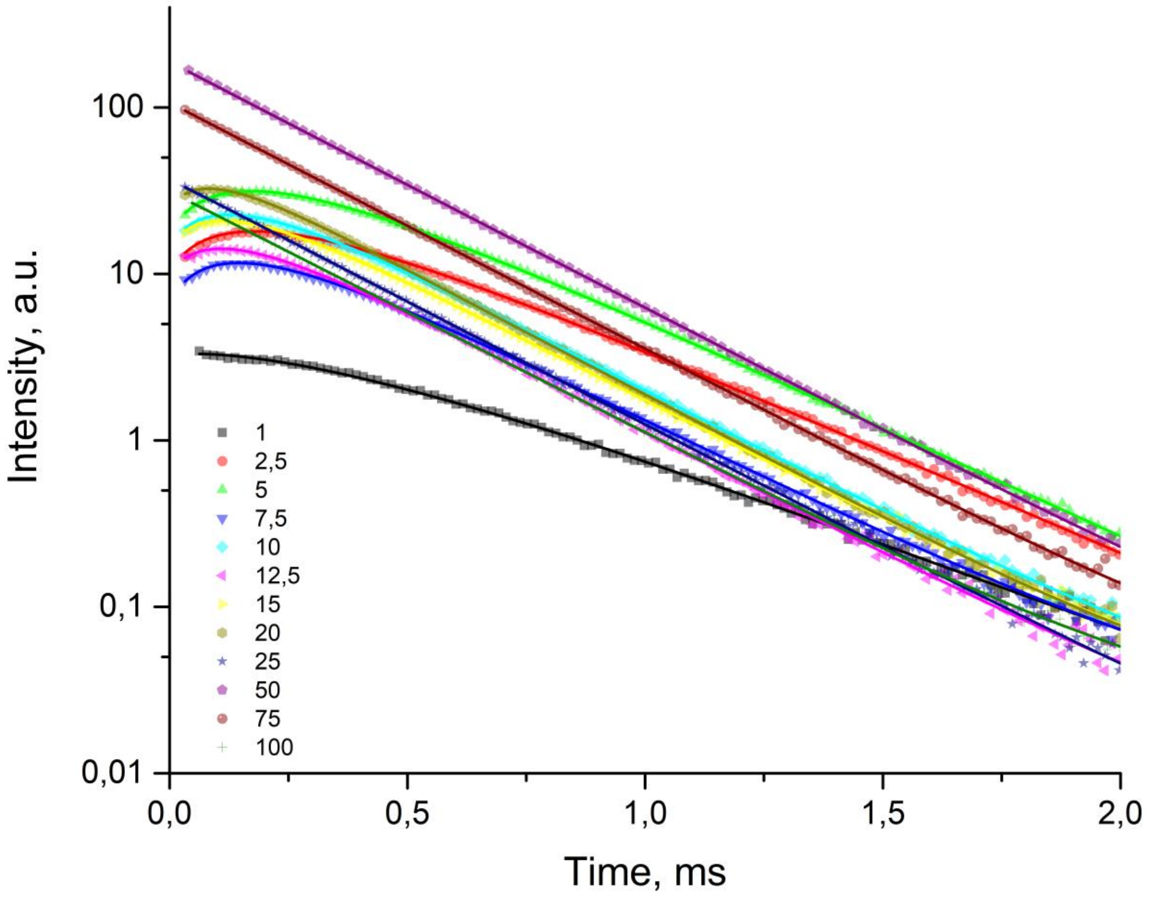

Figure SI13. Eu^{3+} decay curves for for $Eu_xTb_{1-x}L_3$; λ_{EX}=350 nm, λ_{EM}=700 nm.

Table SI7. Eu^{3+} luminescence fitting parameters for $Eu_xTb_{1-x}L_3$

% Eu	Equation: y0+A1*exp(-x/t1)-A2*exp(-x/t2)	Value	Standart error
1	Reduced Chi-Sqr	2.27E-04	
1	Adj. R-Square	0.99986	
1	A1	148.1377	6214.554
1	A2	144.8623	6214.548
1	y0	0.0067	0.00239
1	t1	0.33913	0.18372
1	t2	0.33044	0.18508
1	k1	3.0332	
1	k2	3.0263	
2.5	Reduced Chi-Sqr	1.27E-04	
2.5	Adj. R-Square	0.99999	
2.5	A1	60.35085	0.98515
2.5	A2	49.90638	0.87819
2.5	y0	-0.00183	0.0017
2.5	t1	0.35422	0.00147
2.5	t2	0.17915	0.00205
2.5	k1	2.8231	
2.5	k2	5.5819	
5	Reduced Chi-Sqr	1.02E-04	
5	Adj. R-Square	0.99999	
5	A1	108.5442	1.47104
5	A2	90.94233	1.29326
5	y0	0.00173	0.00156
5	t1	0.33251	0.00105
5	t2	0.17089	0.00165
5	k1	3.0074	
5	k2	5.8517	
7.5	Reduced Chi-Sqr	8.41E-05	
7.5	Adj. R-Square	0.99999	
7.5	A1	33.3927	0.37915
7.5	A2	26.68297	0.31222
7.5	y0	0.0213	8.21E-04
7.5	t1	0.30933	9.82E-04
7.5	t2	0.13714	0.00157
7.5	k1	3.2328	
7.5	k2	7.2918	
10	Reduced Chi-Sqr	7.77E-05	
10	Adj. R-Square	0.99999	
10	A1	59.45618	0.50083
10	A2	44.88323	0.40517
10	y0	0.02033	7.88E-04

10	t1	0.29433	6.68E-04
10	t2	0.12268	0.00135
10	k1	3.3975	
10	k2	8.1513	
12.5	Reduced Chi-Sqr	1.31E-04	
12.5	Adj. R-Square	0.99999	
12.5	A1	32.87276	0.34411
12.5	A2	22.7497	0.28049
12.5	y0	0.0088	9.03E-04
12.5	t1	0.29522	9.35E-04
12.5	t2	0.11128	0.0019
12.5	k1	3.3873	
12.5	k2	8.9863	
15	Reduced Chi-Sqr	8.74E-05	
15	Adj. R-Square	0.99998	
15	A1	49.24294	0.38801
15	A2	34.50976	0.31942
15	y0	0.01735	8.09E-04
15	t1	0.29887	6.80E-04
15	t2	0.11452	0.00145
15	k1	3.3459	
15	k2	8.7321	
20	Reduced Chi-Sqr	7.22E-05	
20	Adj. R-Square	0.99999	
20	A1	60.09556	0.29222
20	A2	35.10217	0.3845
20	y0	0.01923	6.80E-04
20	t1	0.2881	4.30E-04
20	t2	0.08064	0.00137
20	k1	3.4710	
20	k2	12.4008	
25	Reduced Chi-Sqr	0.00195	
25	Adj. R-Square	0.99996	
25	A1	37.44035	0
25	A2	0.5	0
25	y0	0.00436	0
25	t1	0.2938	0
25	t2	0.0674	0
25	k1	3.4037	
25	k2	14.8368	
50	Reduced Chi-Sqr	7.01E-05	
50	Adj. R-Square	0.99999	
50	A1	188.2029	0.56642
50	A2	0.5	2100.604

50	y0	0.02177	9.70E-04
50	t1	0.29378	2.70E-04
50	t2	0.0084	7.97528
50	k1	3.4039	
50	k2	119.0476	
75	Reduced Chi-Sqr	4.74E-05	
75	Adj. R-Square	0.99999	
75	A1	106.9556	0.22718
75	A2	0.5	0
75	y0	0.02183	5.89E-04
75	t1	0.29327	2.06E-04
75	t2	9.14E-04	0
75	k1	3.4098	
75	k2	1094.0919	

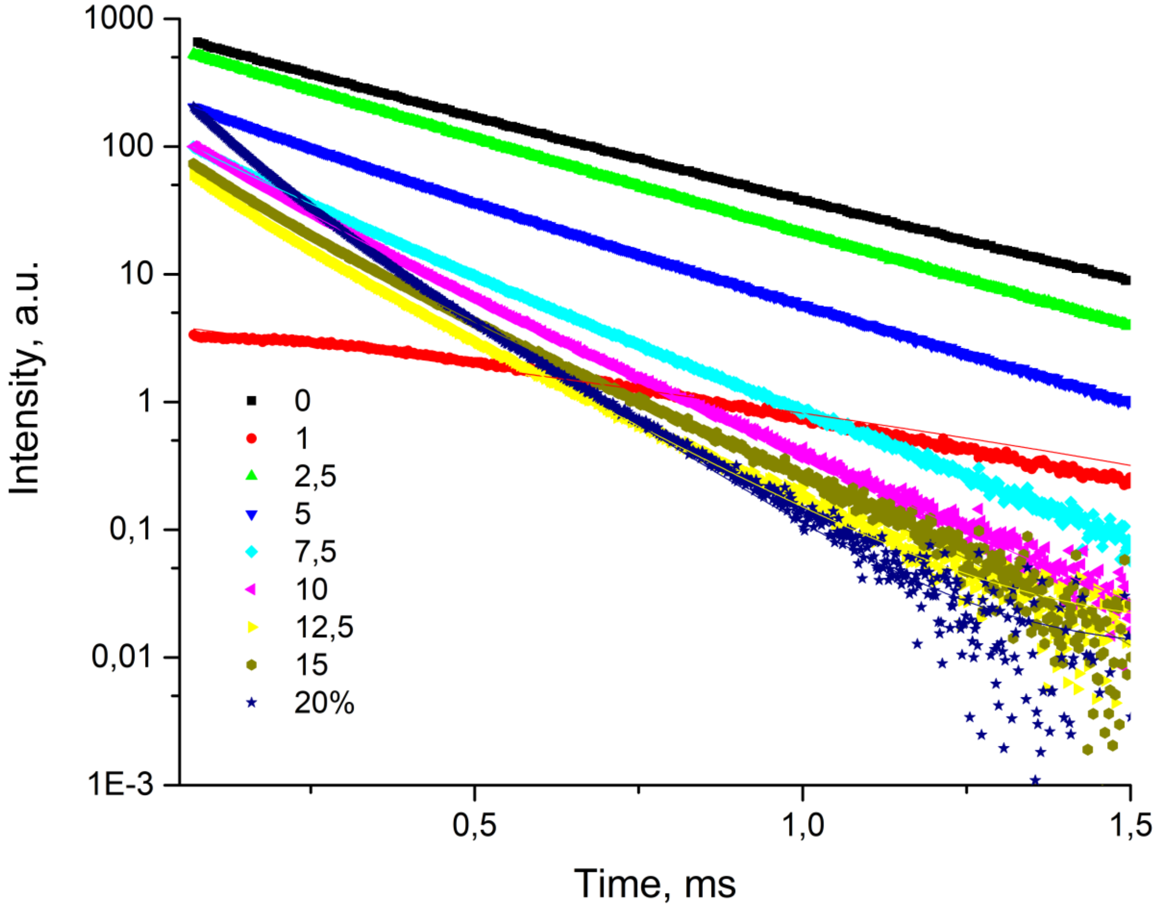

Figure SI14. Tb^{3+} decay curves for for $Eu_xTb_{1-x}L_3$; λ_{EX}=350 nm, λ_{EM}=545 nm.

Table SI8. Tb^{3+} luminescence fitting parameters for $Eu_xTb_{1-x}L_3$

% Eu	Equation : y = A1*exp(-x/t1) + y0	Value	Standart error
0	Reduced Chi-Sqr	2,69135E-1	
0	Adj. R-Square	0,9998	
0	y0	0,56947	0,06047
0	A1	820,32802	0,58271
0	t1	0,31759	2,90255E-4
0	k	3,14868	0,00288
1	Reduced Chi-Sqr	0,01249	
1	Adj. R-Square	0,98805	
1	y0	-0,11705	0,00772
1	A1	4,29164	0,01695
1	t1	0,65636	0,00569
1	k	1,52354	0,0132
2.5	Reduced Chi-Sqr	3,62351	
2.5	Adj. R-Square	0,99981	
2.5	y0	1,27229	0,09105
2.5	A1	677,53819	0,39534
2.5	t1	0,27922	2,64468E-4
2.5	k	3,58136	0,00339
5	Reduced Chi-Sqr	0,62688	
5	Adj. R-Square	0,99967	
5	y0	0,41894	0,03078
5	A1	272,28659	0,23709
5	t1	0,24563	2,89912E-4
5	k	4,07111	0,0048
7.5	Reduced Chi-Sqr	0,66246	
7.5	Adj. R-Square	0,99889	
7.5	y0	0,37357	0,03211
7.5	A1	150,90268	0,24288
7.5	t1	0,17278	3,75647E-4
7.5	k	5,78765	0,01258
10	Reduced Chi-Sqr	0,79236	
10	Adj. R-Square	0,99866	
10	y0	0,35955	0,03356
10	A1	168,51072	0,3135
10	t1	0,14221	3,36402E-4
10	k	7,03197	0,01663
12.5	Reduced Chi-Sqr	0,58873	
12.5	Adj. R-Square	0,99725	
12.5	y0	0,26096	0,02827
12.5	A1	109,48784	0,30237

12.5	t1	0,12566	4,2486E-4
12.5	k	7,9581	0,02691
15	Reduced Chi-Sqr	0,33214	
15	Adj. R-Square	0,99891	
15	y0	0,27593	0,02152
15	A1	124,70506	0,21199
15	t1	0,13546	2,88473E-4
15	k	7,38246	0,01572
20	Reduced Chi-Sqr	3,21667	
20	Adj. R-Square	0,99862	
20	y0	0,6899	0,06325
20	A1	450,30836	0,96644
20	t1	0,09198	2,18713E-4
20	k	10,87222	0,02585

Table SI9. Calculated Tb^{3+} to Eu^{3+} energy transfer rate constants for $Eu_xTb_{1-x}L_3$ compounds

Compound	$k_{ET} = k_1 - k_{Tb}$. k_1 is calculated from Eu^{3+} decay	$k_{ET} = k_1 - k_{Tb}$. k_1 is calculated from Tb^{3+} decay
$Eu_{0.01}Tb_{0.99}L_3$	0.37	0.18
$Eu_{0.025}Tb_{0.975}L_3$	2.95	0.95
$Eu_{0.05}Tb_{0.95}L_3$	3.10	1.32
$Eu_{0.075}Tb_{0.925}L_3$	4.57	3.07
$Eu_{0.1}Tb_{0.9}L_3$	5.47	4.35
$Eu_{0.125}Tb_{0.875}L_3$	6.13	5.10
$Eu_{0.15}Tb_{0.85}L_3$	6.09	4.74
$Eu_{0.2}Tb_{0.8}L_3$	9.75	8.22
$Eu_{0.25}Tb_{0.75}L_3$	12.07	n/a
